# Multitissue Circadian Proteome Atlas of WT and *Per1*^−/−^/*Per2*^−/−^ Mice

**DOI:** 10.1016/j.mcpro.2023.100675

**Published:** 2023-11-07

**Authors:** Liujia Qian, Yue Gu, Qiaocheng Zhai, Zhangzhi Xue, Youqi Liu, Sainan Li, Yizhun Zeng, Rui Sun, Qiushi Zhang, Xue Cai, Weigang Ge, Zhen Dong, Huanhuan Gao, Yan Zhou, Yi Zhu, Ying Xu, Tiannan Guo

**Affiliations:** 1 Westlake Center for Intelligent Proteomics, Westlake Laboratory of Life Sciences and Biomedicine, Hangzhou, Zhejiang Province, China; 2 Institute of Basic Medical Sciences, Westlake Institute for Advanced Study, Hangzhou, Zhejiang Province, China; 3 Research Center for Industries of the Future, Westlake University, Hangzhou, Zhejiang, China; 4 Jiangsu Key Laboratory of Neuropsychiatric Diseases and Cambridge-Suda Genomic Resource Center, Soochow University, Suzhou, Jiangsu Province, China; 5 Westlake Omics (Hangzhou) Biotechnology Co, Ltd, Hangzhou, Zhejiang Province, China

**Keywords:** circadian rhythm, proteomics, PERIOD, mouse, multi-tissue, SCN, knock-out, TMT, tissue specificity, anticipation, synchronization, nucleotide excision repair, intertissue correlation, GABA, chronochemotherapy

## Abstract

The molecular basis of circadian rhythm, driven by core clock genes such as *Per1*/*2*, has been investigated on the transcriptome level, but not comprehensively on the proteome level. Here we quantified over 11,000 proteins expressed in eight types of tissues over 46 h with an interval of 2 h, using WT and *Per1*/*Per2* double knockout mouse models. The multitissue circadian proteome landscape of WT mice shows tissue-specific patterns and reflects circadian anticipatory phenomena, which are less obvious on the transcript level. In most peripheral tissues of double knockout mice, reduced protein cyclers are identified when compared with those in WT mice. In addition, PER1/2 contributes to controlling the anticipation of the circadian rhythm, modulating tissue-specific cyclers as well as key pathways including nucleotide excision repair. Severe intertissue temporal dissonance of circadian proteome has been observed in the absence of *Per1* and *Per2*. The γ-aminobutyric acid might modulate some of these temporally correlated cyclers in WT mice. Our study deepens our understanding of rhythmic proteins across multiple tissues and provides valuable insights into chronochemotherapy. The data are accessible at https://prot-rhythm.prottalks.com/.

## Highlights

• 11,651 proteins are quantified in multitissue circadian proteome atlas.
• Proteome rhythm reflects circadian anticipatory phenomena.
• Tissue-specific protein cyclers in the WT mice are mainly PER-dependent.
• Diminished oscillations in nucleotide excision repair in DKO mice.

## Graphical Abstract

**Figure:**
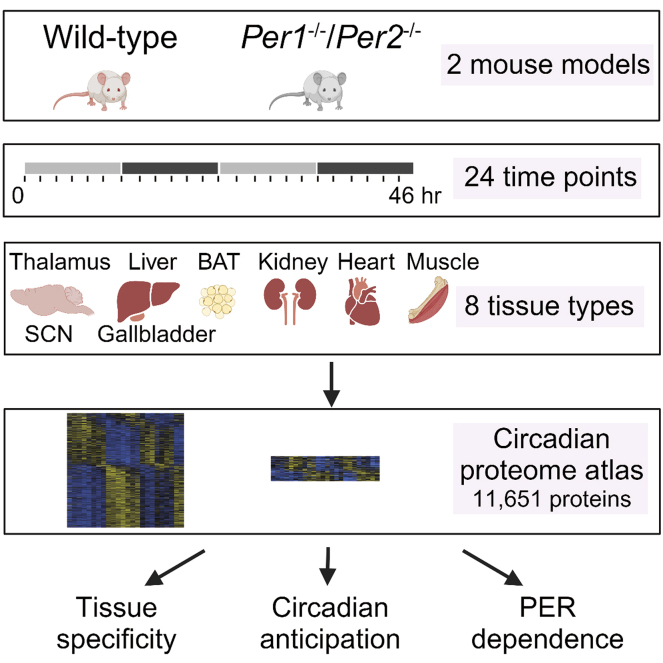

## In Brief

A circadian proteome landscape is established with eight tissue types from WT and *Per1/2* DKO mouse models over a 46-h period with 2-h intervals, characterizing 11,651 proteins. The analysis unveiled tissue-specific patterns and emphasized the role of circadian anticipatory phenomena in WT mice, not as evident at the transcript level. PER1/2 was found to influence circadian anticipation, tissue-specific cycling, and critical pathways such as nucleotide excision repair. This study deepens our comprehension of rhythmic proteins and offers insights into chronochemotherapy.

In mammals, physical, mental, and behavioral activities usually exhibit a 24-h oscillation pattern, which is called the circadian rhythm. The suprachiasmatic nuclei (SCN) in the brain not only maintains its own circadian rhythm but also functions as the master pacemaker to synchronize the rhythm of peripheral tissues in both day-night (*i.e.* light-dark, LD) and constant darkness (*i.e.* dark-dark, DD) conditions (1).

The major molecular basis for circadian rhythm is an autoregulatory transcriptional-translational feedback loop (TTFL), which is expressed in almost every cell. In the TTFL, two transcriptional factors, namely CLOCK and BMAL1, form a heterodimer that can initiate transcription of multiple target genes containing a specific DNA sequence called E-box. Two major groups of these target genes are PERIOD (*Per1*, *Per2,* and *Per3*) and cryptochrome (*Cry1* and *Cry2*) (2). PERs and CRYs form a protein complex, which then translocates to the nucleus and represses the CLOCK-BMAL1 complex-induced transcription, thereby establishing the negative feedback loop (2). In core clock gene KO models, such as *Bmal1*^*−/−*^ mice, *Per1*^*−/−*^; *Per2*^*−/−*^ *mice,* and *Cry1*^*−/−*^; *Cry2*^*−/−*^ mice, locomotor rhythmicity has been reported to be abolished in DD (3, 4, 5). However, retained oscillations in transcripts, proteins, and protein phosphorylation are still observed in *ex vivo* skin fibroblasts and liver slices from *Bmal1*^*−/−*^ mice (6). This sustained rhythmicity in molecules may be mediated by E26 transformation-specific factors and redox oscillations (6). In addition, more rhythmic proteins with higher amplitude are identified in the *ex vivo* lung fibroblasts from *Cry1*^*−/−*^; *Cry2*^*−/−*^ mice than that in the WT mice, probably due to CRYs’ function on protein homeostasis (7). In mouse liver, the daily oscillation in key mitochondrial enzymes and their substrates has been reported to be dependent on the PERIOD proteins (8). However, the role of PERIOD in the circadian proteome, especially in multiple organs and tissues *in vivo*, remains elusive.

Although TTFL are ubiquitous and the SCN can synchronize the peripheral tissues, the number and phase of circadian cyclers are highly tissue-specific at the transcriptional level. Zhang *et al.* analyzed the circadian transcriptome of 12 mouse organs and found that, while the proportion of rhythmic transcripts varied from 3% (in the hypothalamus) to 16% (in the liver) (9), few transcripts were cyclic in more than one organ. A recent diurnal transcriptome study of 64 baboons’ tissues also observed tissue-specific rhythmic expression patterns (10). These results underscore the importance to profile the circadian molecular landscapes across the multiple tissues.

The molecular basis of circadian rhythm driven by TTFL has been extensively investigated on the transcriptome level (9, 10, 11, 12, 13, 14, 15). However, the mRNA level often does not agree with its respective protein level. In particular, the short-term dynamics of proteins are heavily modulated by mRNA-independent mechanisms (16). Recent proteome studies have reported that 20% (17) to 50% (18) of these oscillating proteins are coded by nonrhythmic transcripts, while up to 80% of rhythmic nuclear proteins are encoded by nonrhythmic genes (19). As for the peak phase of the rhythmic genes at both protein and transcript levels, more than six-hour time lag has been identified in the protein cyclers than transcript cyclers. The circadian proteome has been studied in various tissues, such as the SCN (20), hippocampus (21), macrophages (22), cartilage (23), and kidney (24). However, the existing evidence is primarily limited to individual tissues. Nevertheless, these studies collectively emphasize the significant role of protein rhythms in circadian research.

Advanced proteomics technologies now enable the deep, fast, and efficient proteomic profiling of biospecimens (25, 26, 27, 28, 29). However, little is known about how proteins contribute to the spatiotemporal mechanisms orchestrating multitissue circadian rhythms. Here, we report a mouse circadian proteome atlas derived from WT and *Per1*^−/−^; *Per2*^−/−^ double knockout (DKO) mice housed under constant darkness and *ad libitum* feeding. Samples from eight different tissues were collected every 2 h over 2 days and analyzed with tandem mass tag (TMT)-based proteomics. From over 11,000 proteins quantified in the eight tissues, we identified 4749 and 1973 cycling proteins from the WT and DKO mice, respectively. This rich proteomic resource (https://prot-rhythm.prottalks.com/) allows in-depth understanding of the molecular basis of circadian rhythm on protein level and the molecular machineries modulated by PER1/2.

## Experimental Procedures

### Ethics Statement

All the animal procedures were approved by the Animal Care and Use Committee of CAMBRIDGE-SUDA Genomic Resource Center, Soochow University (YX-2019-4).

### Animals and Tissue Collection

Six-week-old male WT C57BL/6J mice were purchased from Vital River. Mice with mutations targeting the *Per1* and *Per2* genes were generated as previously described (4, 30). The locomotor activities were recorded by the ClockLab (Actimetrics). The dynamic expression of core clock genes was assayed by Western blot to check the arrhythmicity in the DKO mouse model. The *Per1* and *Per2* mutant mice were bred with WT C57BL/6J mice for at least 10 generations. *Per1* and *Per2* DKO mice were generated by homozygote mating. All mice were fed a standard chow diet (ShooBree specific pathogen-free Mice Diet, including 28% protein, 13% fat, and 57% carbohydrates). Before the tissues collection, all our mice were synchronized under cycles of 12 h of light and 12 h of dark for at least 1 week. WT and DKO mice were then released into constant darkness. Eight tissue types (thalamus (TLM), SCN-containing region, liver, gallbladder with bile (GBD), brown adipose tissue (BAT), kidney, heart, and gastrocnemius muscle) were collected every 2 h for 2 days to obtain 24 time points (under constant darkness between T0 and T46). The heart we dissected contains both ventricle and atrium; the gallbladder dissected containing bile to characterize the rhythmic proteins in bile. Before tissue sampling, each mouse was injected with pentobarbital sodium; heart perfusion with PBS was performed. All samples were collected on ice in 10 min and then immediately frozen in liquid nitrogen. The collected brain tissue was dipped in ice-cold PBS for 1 min. A 1 mm brain slice was then obtained using stainless brain matrices (68707, Shenzhen RWD Life Science Co, Ltd), and SCN-containing region was isolated with a 1.5 mm tissue punch (Integra Miltex). To avoid the biological variation during SCN-containing region dissection, all samples were collected by the same operator who has been professionally trained.

### Experimental Design and Statistical Rationale

In this study, we aimed to conduct in-depth proteomics analysis to investigate protein cyclers with high temporal resolution. We utilized guidelines for genome-scale analysis of biological rhythms (31) to design our experimental approach: (1) biological replicates: (a) to account for variability, we collected two complete cycles of data as recommended by the guidelines; (b) tissue samples were collected at 2-h intervals across the two complete cycles to enhance temporal resolution; (c) three time points in each type of tissue were randomly selected, and biological replicates were collected from two mice at each of these time points (supplemental Table S1); (2) technical replicates: to assess the reproducibility of the proteomic data, TMT-labeled proteome data were analyzed twice from the same peptide sample collected at three arbitrarily selected time points, serving as technical replicates (supplemental Table S1). The high reproducibility observed between biological and technical replicates validated the reliability of the proteomic data.

Three factors were taken into consideration for statistical rationale: (1) Temporal resolution: To capture fine-scale rhythmic patterns, we adopted a 2-h sampling resolution, as suggested by the guidelines (31). This resolution offers improved accuracy in detecting circadian rhythms compared to traditional 4-h sampling intervals. (2) Exploratory analyses: Our primary objective was to explore rhythmic patterns rather than conduct formal hypothesis testing. For exploratory analyses, even with a single replicate sample at each time point, we employed MetaCycle to calculate *p*-values. This approach allowed us to assess the presence of rhythmicity in the data. (3) Cost optimization: To optimize experimental expenses, we collected one sample for each tissue type at the remaining time points, as the high temporal resolution from the two complete cycles provided sufficient information for our exploratory analyses.

In summary, we collected two complete cycles of data as biological replicates and used a 2-h sampling resolution to enhance temporal resolution. Exploratory analyses were conducted using MetaCycle, allowing the calculation of *p*-values even with a single replicate at each time point. The high reproducibility observed between biological and technical replicates validated the reliability of our proteomic data.

### Locomotor Activity

Two-month-old male WT and *Per1*/*Per2* DKO mice were individually housed in cages equipped with wheels and were synchronized for 1 week on a LD cycle with lights on at 8 AM and off at 8 PM. Mice were then released into constant darkness for 4 weeks. Mice had free access to water and food during the experiment. Wheel running was recorded and analyzed using the ClockLab.

### Western Blot

Mice were sacrificed by cervical dislocation at 0, 4, 8, 12, 16, 20, and 24 h after transferred into constant darkness, respectively. Hundred milligrams of liver samples were collected from each mouse and immediately transferred to −80 °C for storage. After all samples were collected, the liver samples were lysed on ice with 1 ml of protein lysis buffer. The protein supernatant was collected by centrifugation at 12,000 rpm for 10 min at 4 °C. The protein samples were then subjected to denaturing SDS-PAGE and transferred onto PVDF membranes. After blocking with 5% milk at room temperature for 30 min, the membranes were incubated with primary antibodies (anti-ACTIN: sigma A5441, 1:5000; anti-BMAL1: 1:1000; anti-CLOCK: CST 5157S, 1:500; anti-CRY1: 1:1000; anti-PER1: MBL PM091, 1:1000; anti-PER2: MBL PM083, 1:1000) overnight at 4 °C. Rabbit polyclonal BMAL1 and CRY1 antibodies were generated by Signalway Antibody using synthetic peptides of antigen BMAL1: CSSSILGENPHIGIDMIDNDQGSSSPSNDEA; CRY1: CSQGSGILHYAHGDSQQ THSLKQGRSSAGTG and validated in KO mouse models. Incubate with secondary antibodies (Goat anti-mouse IgG-HRP: sc-2005, 1:10,000; Goat anti-rabbit IgG-HRP: sc-2004, 1:10,000) at room temperature for 1 h in the next day and visualize the protein of interest using a multifunctional imaging system (Chemiscope 6300).

### TMT-Based Proteomics Profiling

About half of the fresh frozen TLM, liver, BAT, kidney, heart, and gastrocnemius muscle samples were homogenized for 30 s with two mill beads of 3 mm diameter (G0103-200G, Servicebio) in a lysis buffer (6M urea (Sigma), 2M thiourea (Sigma), protease inhibitor (cOmplete Tablets EASYpack, Roche)) using a tissue homogenizer (Speedmill PLUS, Analytik-Jena AG). The GBD were homogenized together since it was impractical to isolate them reproducibly. About 1 to 2 mg tissue lysates were subject to proteolysis. In the case of SCN-containing region, the entire tissue (around 0.5 mg) was used for peptide generation. Peptide samples of all eight types of tissues were generated using accelerated pressure cycling technology–assisted sample preparation as previously described (32, 33, 34). Briefly, the samples were lysed in the lysis buffer, reduced by Tris (2 carboxyethyl)phosphine (Sigma), and alkylated by iodoacetamide (Sigma) in pressure cycling technology. The samples’ lysates were then digested in a mixture of Lys-C and trypsin (Hualishi Tech. Ltd). After digestion quenching with TFA, peptides were desalted using SOLAμTM solid-phase extraction well plates (Thermo Fisher Scientific). For TLM, liver, BAT, GBD, SCN-containing region, and gastrocnemius muscle samples, 30 μg peptides from each of the 288 tissue samples, 36 biological replicates, and 36 tissue-specific pooled controls (supplemental Table S1) were redissolved with 30 μl 100 mM TEAB, then 9 μl of 20 μg/μl TMT 10plex (Thermo Fisher Scientific) in anhydrous acetonitrile and additional 6 μl of anhydrous acetonitrile was added into each sample for TMT labeling. Vortex and incubate at 25 °C and 600 rpm for 60 min on the Thermo Shaker Incubator. Then, labeling efficiency was evaluated. Then the labeling reaction was quenched by 3 μl of 5% hydroxylamine if the labeling efficiency reaches K-term >95% and N-term >95%. For the heart and kidney samples, 7 μg peptides from each of the 96 tissue samples, 12 biological replicates, 12 technical replicates, and eight tissue-specific pooled controls (supplemental Table S1) were redissolved with 2.8 μl 100 mM TEAB, then 1.4 μl of 20 μg/μl TMTpro 16plex (Thermo Fisher Scientific) in anhydrous acetonitrile. Additional 0.5 μl of anhydrous acetonitrile was added into each sample for TMT labeling. After vortex and incubation at 25 °C and 600 rpm for 60 min in the shaker, the reaction was quenched by 0.3 μl of 5% hydroxylamine following the same requirements for labeling efficiency. After the labeling reaction, the combined peptides were fractionated (using 120 min or 135 min liquid chromatography (LC) gradient, ramping from 5 to 34% acetonitrile (ACN) in 10 mM ammonia (pH = 10), at a flow rate of 1 ml/min) into 120 or 135 fractions (details are provided in supplemental Table S1) on a Thermo Ultimate Dionex 3000 (Thermo Fisher Scientific) with an XBridge Peptide BEH C18 column (300 Å, 5 μm × 4.6 mm × 250 mm) (Waters). The 40 to 45 fractions’ combinations (supplemental Table S1) were dried in a vacuum and then redissolved in 2% ACN/0.1% formic acid. The fractionated peptide samples were analyzed using a Q Exactive HF-X or an HF hybrid Quadrupole-Orbitrap (Thermo Fisher Scientific). For each injection, peptides were loaded into a precolumn (3 μm, 100 Å, 20 mm∗75 μm internal diameter (i.d.)) at a flow rate of 6 μl/min and then into the analytical column (1.9 μm, 120 Å, 150 mm∗75 μm i.d.) at a flow rate of 300 nl/min using a 45 min LC gradient. The MS1 resolution was 60,000 (at 200 m/z), the scan range was 350 to 1800 m/z, the automatic gain control target was 3E6, and the maximum ion injection time (IT) was 50 ms. In the MS/MS experiment, the automatic gain control target was 2E5, the maximum ion injection time was 100 ms, and the isolation window was 0.7 m/z. For SCN-containing region, gastrocnemius muscle, GBD, BAT, heart, and liver, the peptide samples were analyzed by a Q Exactive HF hybrid Quadrupole-Orbitrap with an MS2 resolution of 60,000 (at 200 m/z). The TLM and kidney peptide samples were analyzed by a Q Exactive HF-X hybrid Quadrupole-Orbitrap with an MS2 resolution of 45,000 (200 m/z).

### RNA-Sequencing

Total RNA was extracted from liver samples using Trizol Reagent (Invitrogen) according to the manufacturer’s instructions. The mRNA was purified by oligo(dT)-attached magnetic beads and fragmented into small pieces. The complementary DNA (cDNA) fragments were generated by random hexamer-primed reverse transcription and A-Tailing Mix and RNA Index Adapters for end repair. The quality of the amplified and purified cDNA was determined on the Agilent Technologies 2100 bioanalyzer. Then, the double stranded cDNA products were denatured and circularized into single strand circle DNA to construct the final library. DNA nanoballs with more than 300 copies for each molecule were generated by amplification with phi29. RNA-seq was performed on the BGIseq500 platform (BGI-shenzhen). RNA gene expression profiles were analyzed using RSEM (version 1.2.8) with UCSC-annotation (version mm10) and normalized by Fragments Per Kilobase of exon model per Million mapped fragments (FPKM).

### Proteomics Data Processing and Analysis

The generated mass spectrometry raw data were analyzed using Proteome Discoverer (Version 2.4.1.15, Thermo Fisher Scientific, https://thermo.flexnetoperations.com/control/thmo/download?element=11324157) and a FASTA file of 17,023 reviewed *Mus musculus* proteins downloaded from the UniProt website on 17 January 2020. For heart and kidney, TMTpro (+304.207 Da) of the C terminus and any N terminus were set as static modifications. For other six remaining tissue types, TMT6plex (+229.163 Da) of C terminus and any N terminus were set as static modifications. The other parameters of Proteome Discoverer were set as in our previous study (28). Briefly, the enzyme to generate peptides was selected as Trypsin and no more than two missed cleavages were permitted. Mass tolerance for precursor ions and fragment ions were set as 10 ppm and 0.02 Da, respectively. Oxidation (M) and acetyl (protein N-terminus) were set as variable modifications and carbamidomethyl (C) as the static modification. The quantified proteins and peptides with false discovery rates both below 1% were subject to further analysis. The final protein expression values in the protein matrix were calculated by the grouped abundance ratio of tissue samples *versus* the tissue’s pooled controls from the same batch (supplemental Table S2).

### Quality Control of the Proteomics Data

We first pooled the peptides of all the samples derived from the same tissue of the WT and the DKO mice: eight types of pooled peptide samples were labeled with one plex of TMT 10plex or TMTpro 16plex in each batch (details were provided in supplemental Table S1) for batch alignment. Next, the mouse liver protein digests for the MS instrument performance evaluation were analyzed every two batches, and an MS buffer A blank (2% ACN/0.1% formal acid) was measured every four LC-MS/MS injections. We also analyzed three biological replicates, randomly selected from all the time points, for each tissue type and technical replicates for the kidney and heart (supplemental Table S1). Finally, we calculated the coefficient of variation using the pooled samples' protein abundance and the protein expression ratio from the technical and the biological replicates for proteome reproducibility (supplemental Fig. S2).

### Selection of Replicate Samples

Metacycle requests the same number of replicate samples; thus, after confirming the high reproducibility between replicate samples, we randomly selected one sample from the designed biological and technical replicates for each time point for rhythmicity analysis (supplemental Table S1).

### Rhythmicity Analysis Using Metacycle

To perform the rhythmicity analysis for two datasets, the proteome matrix of the samples collected during shifting different temporal interval, namely from T0 to T46, from T12 to T46 and from T18 to T46 were used. The proteins with more than seven missing values were filtered out at first. Then, the missing values of the remaining proteins were imputed using a modified version of the last observation carried forward and the next observation carried backward (35). In this approach, if the missing value is at the start or end time point, we imputed it using the nearest time point. Otherwise, the missing value of a specific protein is replaced by the average expression value of the two nearest time points. To evaluate the mRNA cyclers, a previously published transcriptome matrix (9) was downloaded and newly analyzed. The meta2d function of the MetaCycle package (36) was used to analyze both these time-series datasets. MetaCycle package incorporates ARSER, JTK_CYCLE, and Lomb-Scargle to evaluate periodicity in time-series data using an N-version programming method, and meta2d is one of the two functions in this package to analyze data from a single time series (36). A relative amplitude value (rAMP) taken as the ratio between amplitude and baseline was generated by meta2d to compare the amplitude values among genes regardless their different expression levels. The minimum period was set to 23.1, the maximum period was set to 24.9, and the default period for ARS was set to 23.7; the other parameters were left to their default values. To avoid the interference of intrinsic noise during gene expression (37, 38, 39, 40) on circadian proteome, a threshold of 10% rAMP was additionally chosen to define the circadian oscillation of protein cyclers (7). Then, we compared the circadian proteome from T12 to T46 at three different thresholds, namely meta2d_pvalue <0.1 and rAMP ≥0.1, meta2d_pvalue <0.05, and meta2d_BH.Q < 0.2 using MetaCycle (41) (supplemental Table S3). Comparable and robust circadian rhythm was observed in protein cyclers selected by three different thresholds. Protein cyclers defined at threshold of meta2d_BH.Q < 0.2 exhibited a more drastic change dependent on the numbers of protein cyclers (supplemental Table S3). In most tissues, fewer protein cyclers were identified at threshold of meta2d_pvalue <0.1 and rAMP ≥0.1 when compared to those of meta2d_pvalue <0.05. Thus, we defined protein cyclers at a threshold of meta2d_pvalue <0.1 and rAMP ≥0.1. The mRNA cyclers were filtered using meta2d_pvalue <0.05. In addition, we defined as high-rAMP protein cyclers those with meta2d_pvalue <0.1 and rAMP ≥0.3 and as robust protein cyclers those with *p* values <0.01 (calculated using meta2d, JTK and ARS).

### Comparison of Rhythmic Characteristics by CircaCompare

The common cyclers between transcriptome and proteome, as well as between WT and DKO proteome, were identified using MetaCycle as previously mentioned. The differences in phase, mesor, and amplitude of there common cyclers were then compared by the nonlinear cosinor regression tool, CircaCompare (42). Due to the differences in expression levels between mRNA and protein, mesor and amplitude comparisons were not performed between transcriptomic and proteomic data. The cyclers identified as nonrhythmic by CircaCompare were excluded at first. The same cyclers between WT and DKO proteome were defined as Benjamini-Hochberg–adjusted *p* values >0.05 (calculated for difference in phase, mesor, and amplitude), while common cyclers with any of Benjamini-Hochberg–adjusted *p* values <0.05 were regarded as change cyclers.

### Phase Set Enrichment Analysis

The most well-synchronized pathways from both the protein and the mRNA cyclers in the WT mice were enriched using phase set enrichment analysis (43) and selected with Kuiper’s *p* value <0.05 (vs. background), Kuiper’s q-value <0.05 (vs. uniform) (supplemental Table S5). The top five most well-synchronized pathways were then ranked using vector-average magnitude.

**Fig. 1:**
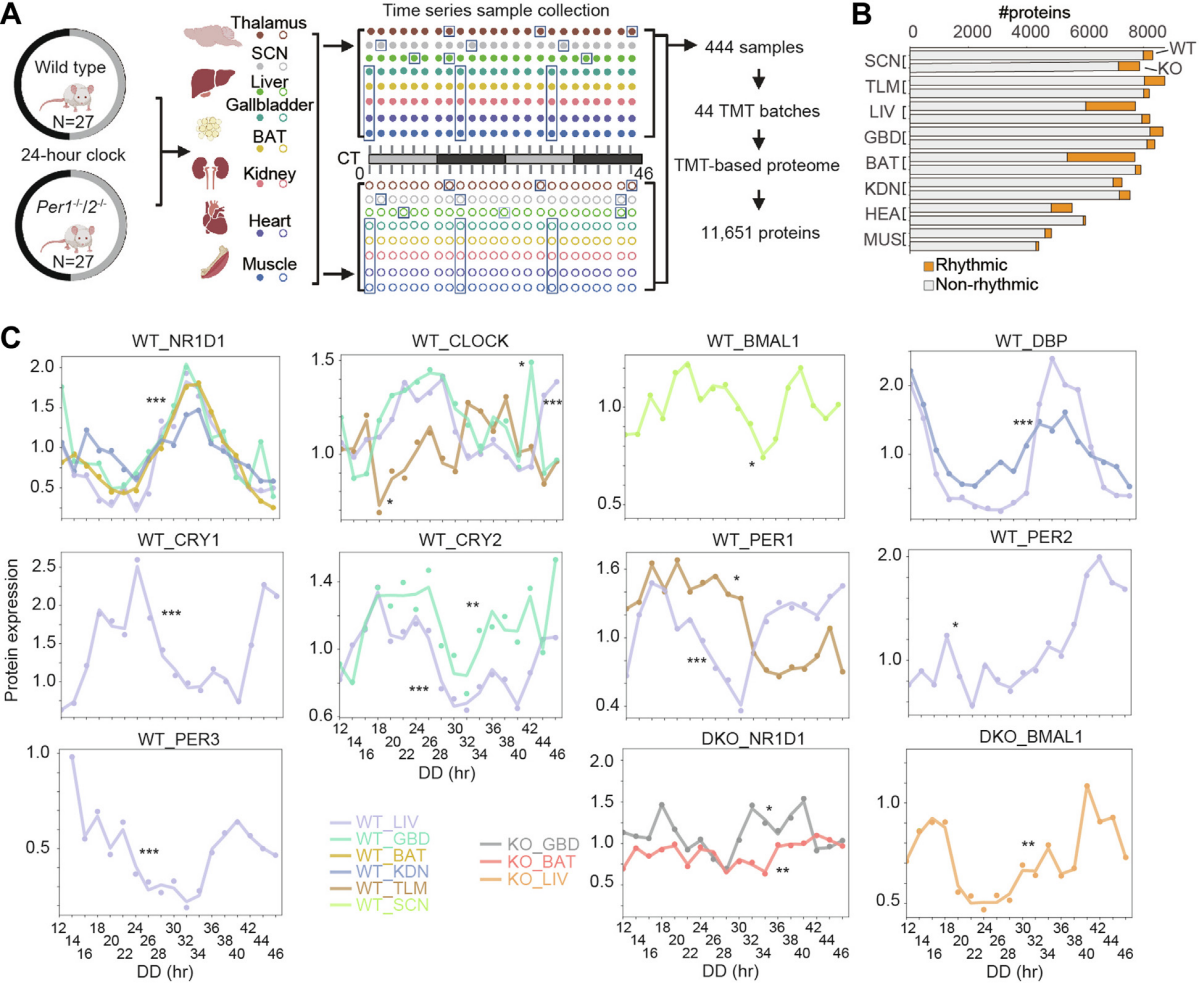
**Proteomics workflow and overview of the multitissue circadian landscape of WT and *Per1***^***−/−***^***/2***^***−/−***^**(DKO) mice.***A*, workflow of the TMT-based quantitative proteomics analysis. Eight tissue types were collected from both WT and DKO mice every 2 h for 2 days (CT0 is the onset of the first *dark cycle*). A total of 488 samples, including 48 biological replicates, 12 technique replicates, and 44 tissue-specific pooled controls, were distributed into 44 batches for proteomics analysis. The replicates are grouped in *blue* boxes. More details are provided in supplemental Table S1. *B*, the bar plot represents the number of quantified proteins and the fraction of rhythmic proteins (*orange*) from each tissue and mouse model. *C*, the time-series curves represent the dynamic expression of the identified core clock proteins from the transcription-translation feedback loop (TTFL). ∗, 0.01 < *p* value <0.1; ∗∗, 0.001 < *p* value <0.01; ∗∗∗, *p* value ≤0.001. SCN indicates the SCN-containing region. DKO, double knockout; SCN, suprachiasmatic nuclei; TMT, tandem mass tag.

**Fig. 2:**
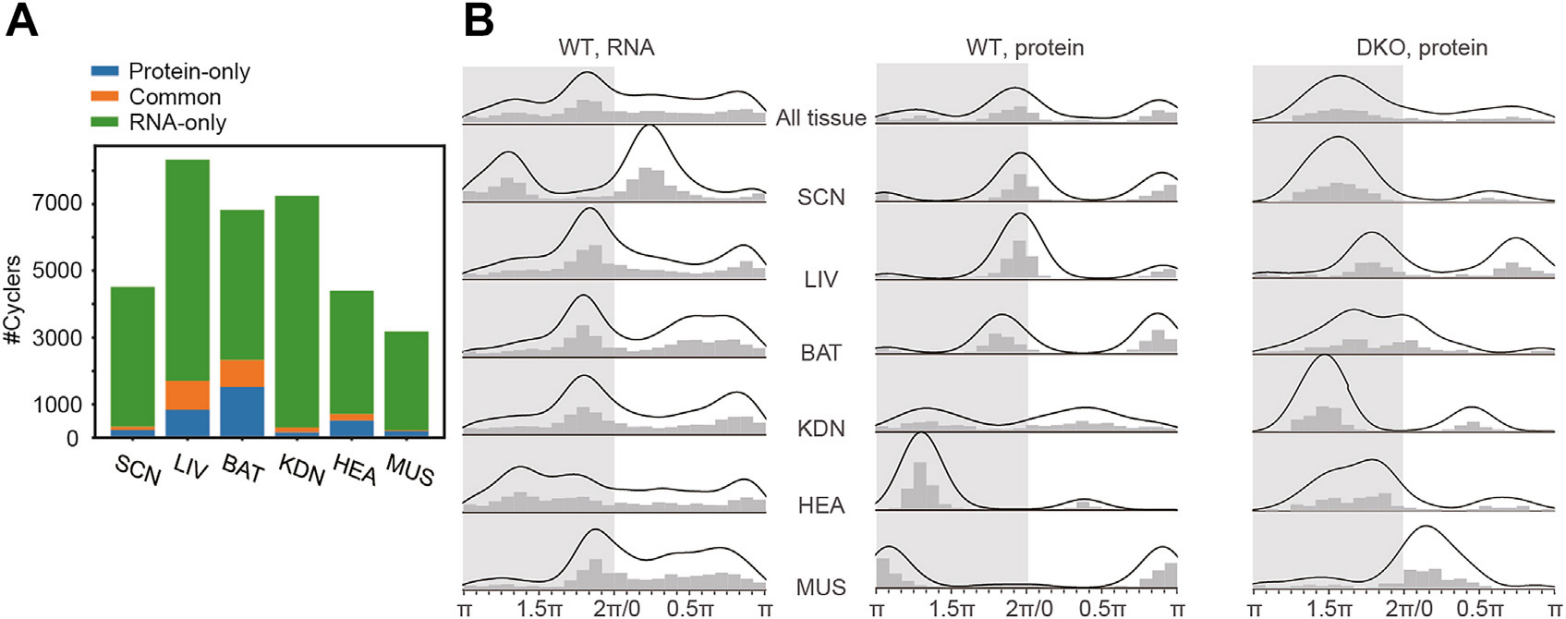
**Comparison of the rhythmic mRNAs and proteins in multiple tissues from WT mice.***A*, the bar plot represents the protein-only cyclers, the mRNA-only cyclers, and the cyclers common to the transcriptome and the proteome. The cycling mRNA data were identified using meta2d (*p* value <0.05) from a previously published dataset. *B*, phase distribution of rhythmic mRNAs and proteins across six tissues in the WT mice and the DKO mice. The uppermost plot represents the phase distribution of cyclers from all six tissues. The Y-axis coordinates of the bar plots in each column are the same. The X-axis represents peak phase, with the values 0 and π denoting T0 and T12, respectively. SCN indicates the SCN-containing region. DKO, double knockout; SCN, suprachiasmatic nuclei.

**Fig. 3:**
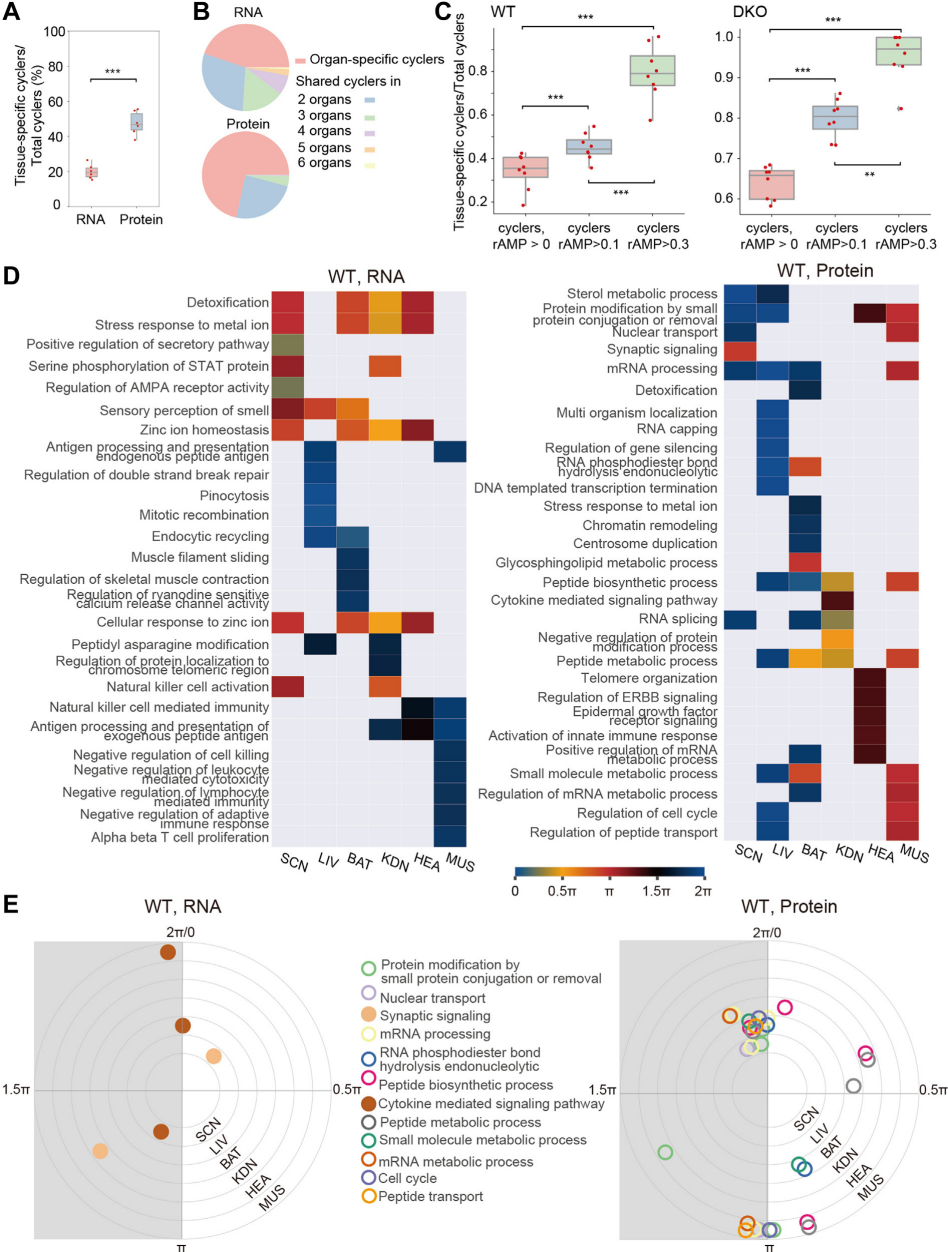
**Tissue specificity of the circadian proteome.***A*, boxplots showing the proportion of tissue-specific cyclers compared to the total number of cyclers in each tissue. The comparison between transcripts and proteins of each tissue was performed using Student’s *t* test. ∗∗∗, *p* value <0.001. *B*, the fractions of tissue-specific and shared cyclers are represented as pie plots, illustrating their proportions relative to the total number of cyclers across six different tissues. *C*, boxplots showing percentages of the tissue-specific cyclers across eight tissues with different cutoffs for rAMP. Each dot represents the percentage of tissue-specific cyclers over total cyclers of each tissue. The comparison between the different cutoff groups was performed using Student’s *t* test. ∗∗∗, *p* value <0.001. *D*, enriched pathways and their phase distributions generated using a phase set enrichment analysis (PSEA) with rhythmic transcripts or proteins from each tissue. The pathways were further filtered by Kuiper’s *p* value <0.05 (*versus* background) and Kuiper’s q-value <0.05 (*versus* uniform), and the top five pathways were selected by vector-average magnitude. *E*, pathways shared by multiple tissues, generated by PSEA and selected for their inversed phases (the phase shift between two tissues was greater than 10 h) among all six tissues. Each ring represents a specific tissue type, while the dots positioned on the rings indicate the enriched pathways. The angle of each dot corresponds to the phases that are enriched by PSEA. SCN indicates the SCN-containing region. rAMP, relative amplitude value; SCN, suprachiasmatic nuclei.

### Dysregulated Proteins Between the WT and the DKO Mice

A Student’s *t* test was performed on the protein matrix of the samples collected from T0 to T46. We excluded the samples with missing values by batch, and the left missing values were imputed to 0.01, which is 50% of the minimum in the whole protein matrix. The dysregulated proteins were selected with the Benjamini-Hochberg–adjusted *p* value <0.05. The Log_2_(fold-change) between the WT and the DKO mice was then calculated using the average protein expression over 2 days.

### Temporal Correlation

We calculated Spearman’s rank correlation coefficients and significances of expression ratios of each overlapped protein cycler from T12 to T46 between each two tissues in the WT and DKO mice. The protein cycler with a temporal correlation *p* value <0.05 was considered as significant.

### Pathways and Networks Analysis

Ingenuity pathway analysis (IPA) was used to perform the pathway enrichment and the upstream regulator analysis for i) the protein cyclers from each tissue of the WT and DKO mice; ii) the protein cyclers with significant temporal intertissue correlation from the WT and the DKO mice. The upstream regulator analysis is based on prior knowledge of expected effects between transcriptional regulators and their target genes. The upstream transcriptional regulators include transcription factor, microRNA, kinase, compound, and drug. For each analysis, two statistical measures, namely an overlap *p*-value and an activation z-score, were computed.

The network analysis of the high rAMP cyclers of the WT and the DKO mice was performed using IPA, and the network with highest scores was visualized. The network analysis of the multitissue-shared cyclers was performed with Cytoscape StringApp (44). The Gene Ontology analysis of the multitissue-shared cyclers and the tissue-specific PER-dependent cyclers was performed with a false discovery rate <0.05, a redundancy cutoff <0.1, and we manually checked the final results.

The network analysis of the robust cyclers was performed using Rcy3 and with Cytoscape (45). Among the clusters of the resulting network, we selected those composed of more than 20 protein cyclers. We then analyzed the enriched pathways of each selected cluster using String (44). After a manual check, the top two most significant pathways were labeled with the same color as the corresponding cluster. In addition, we calculated the cyclers proportion within each tissue and labeled the tissues with a cycler proportion higher than 12.5%.

### Other Statistical Analyses

To compare the tissue-specific and the PER-(in)dependent cyclers, a paired Student’s *t* test was performed. To evaluate the nonrandom associations between the low- or high-rAMP cyclers and (non-) secreted cyclers in the GBD, we performed Fisher’s exact test and calculated the two-sided *p* value.

## Results

### A Multitissue Circadian Proteome Landscape of WT and *Per1*^−/−^; *Per2*^−/−^ Mice

**Figure S1:**
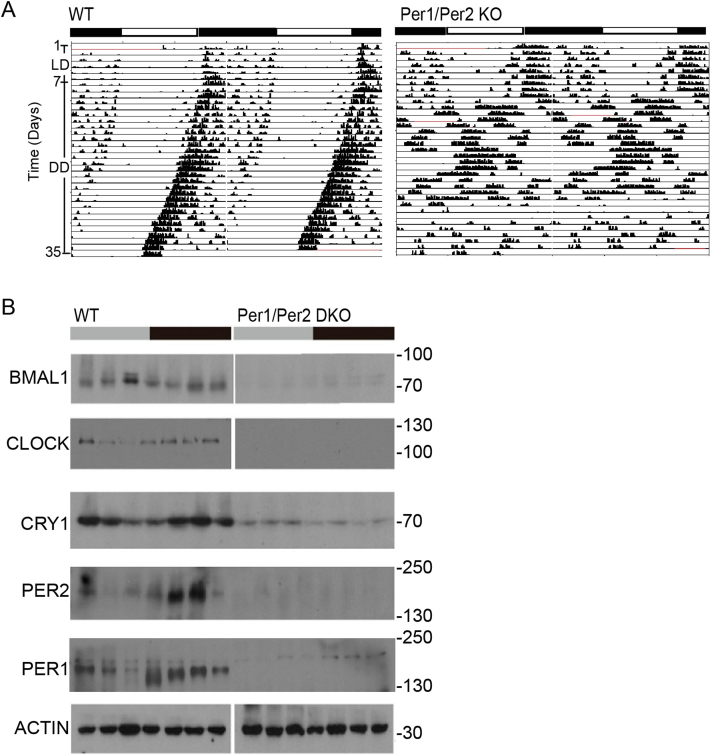
**The characterization of circadian rhythm in WT and DKO mice.***A*, the locomotor activity recorded in the first seven cycles of 12 h of light and 12 h of dark and the following four weeks of the constant dark. *B*, The time-series expression of core clock proteins in liver tissues of WT and DKO mice by Western blot.

To investigate the circadian rhythm at the proteomic level, we first synchronized WT mice on the C57BL/6J background under 12-h light and 12-h dark cycles (LD) for 1 week. Subsequently, the mice were transferred into constant darkness (DD) to commence sampling (Fig. 1*A*). The first time point of sampling was designated as "circadian time 0 (T0)" which corresponded to the precise transition from darkness to light based on the previous LD cycles. We collected samples from eight different tissues, namely SCN-containing region, TLM, liver (LIV), GBD, BAT, kidney (KDN), heart (HEA), and gastrocnemius muscle (MUS), at two-hour intervals over a span of 2 days (Fig. 1*A* and supplemental Table S1). To investigate the role of PERIOD in multitissue proteome dynamics, we synchronized and sampled *Per1*^*−/−*^*; Per2*^*−/−*^ mice under the same experimental scheme as the control group (Fig. 1*A* and supplemental Table S1). In the *Per1*^−/−^; *Per2*^−/−^ mouse model, we have observed the depletion of PER1 and PER2 proteins, the arrhythmic expression of other core clock proteins and loss of rhythm in locomotor activity under constant dark (supplemental Fig. S1).

**Figure S2:**
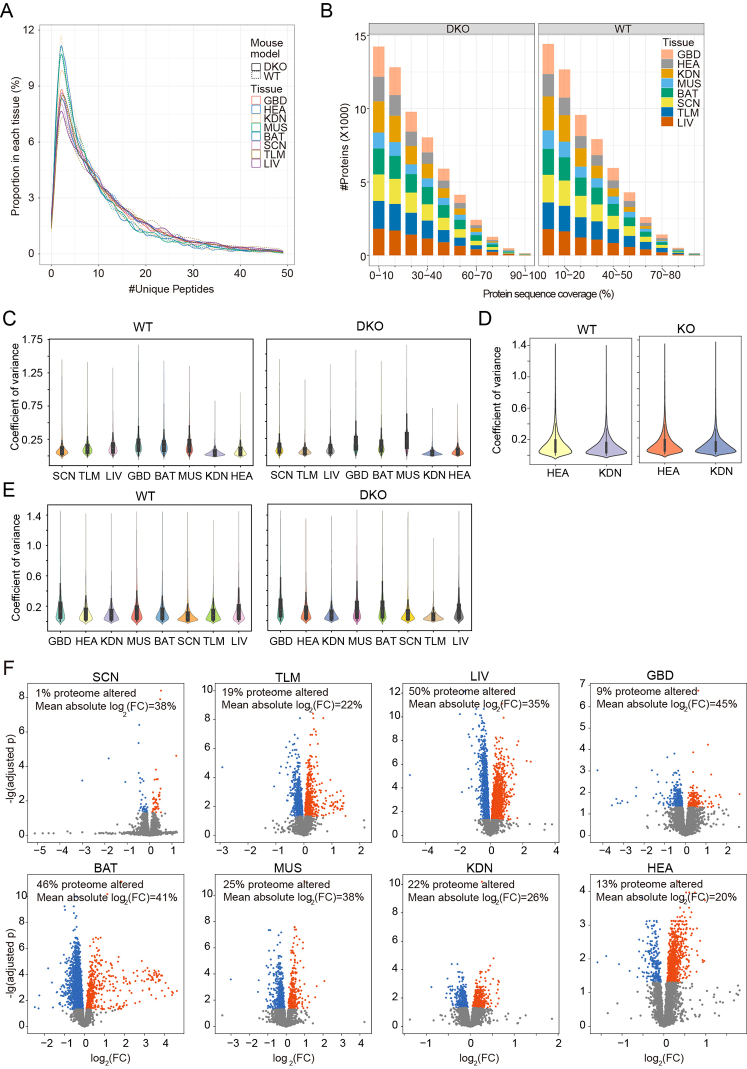
**Quality control for the proteomics analysis, Related to Figure 1.***A*, the density plot represents the distribution of the unique peptide number of quantified proteins in each type of tissue. *B*, the bar plot represents the protein number at each range of protein sequence coverage. The violin plots represent the coefficient of variance between the tissue-specific pooled controls (*C*), the biological (*D*), and the technical (*E*) replicates from each tissue. *F*, the volcano plots represent the dysregulated proteins between the two mice models. The cutoff was established using the Benjamini-Hochberg–adjusted *p* value < 0.05. The proportion of dysregulated proteins in each tissue and the mean absolute log_2_(fold change) of these proteins are indicated in each plot. The upregulated proteins are labeled in red and the downregulated ones in blue. SCN indicates the SCN-containing region.

Using TMT-based proteomics, we quantified 11,651 proteins from 444 peptide samples distributed across 44 batches (Fig. 1*A* and supplemental Table S1). We found that 75.21% of proteins examined identified at least three unique peptides (supplemental Fig. S2*A*). In addition, 54.39% of the proteins exhibited coverage of at least 20% of their protein sequence (supplemental Fig. S2*B*). To assess the quality of our data, we employed 48 biological replicates for three randomly selected time points in each tissue, along with 12 technical replicates and 44 tissue-specific pooled controls derived from all the TMT batches (supplemental Table S1). All the median coefficients of variance of the proteins’ quantification were below 10%: 8.42% for the biological replicates, 8.81% for the technical replicates, and 9.01% for the pooled controls. These results indicate a high degree of reproducibility of our data (supplemental Fig. S2, *C*–*E*). The number of identified proteins varied across tissues, ranging from 4417 (MUS of DKO mice) to 8734 (TLM of WT mice) (Fig. 1*B* and supplemental Table S2).

We next conducted a comparative analysis of the global proteome between the WT and the DKO mice for each tissue (supplemental Fig. S2*F*). Our findings revealed significant dysregulation (Benjamini-Hochberg [B-H]–adjusted *p* value <0.05) in the proteomes of DKO mice, ranging from 1% to 50% of the proteins examined (supplemental Fig. S2*F* and supplemental Table S3). In particular, nearly half of the proteomes of BAT and liver were significantly altered, with an average absolute log_2_(fold change) of 30 to 40% (supplemental Fig. S2*F* and supplemental Table S3). On the other hand, in the SCN-containing region and GBD, although less than 10% of the proteomes were altered, their average absolute log_2_(fold change) was nonetheless as high as that in BAT and liver (supplemental Fig. S2*F* and supplemental Table S3). These results emphasize the significant tissue-specific perturbations in proteomes resulting from the deletion of *Per1* and *Per2*.

### Reduced Protein Cyclers in DKO Mice Across Multiple Peripheral Tissues

**Figure S3:**
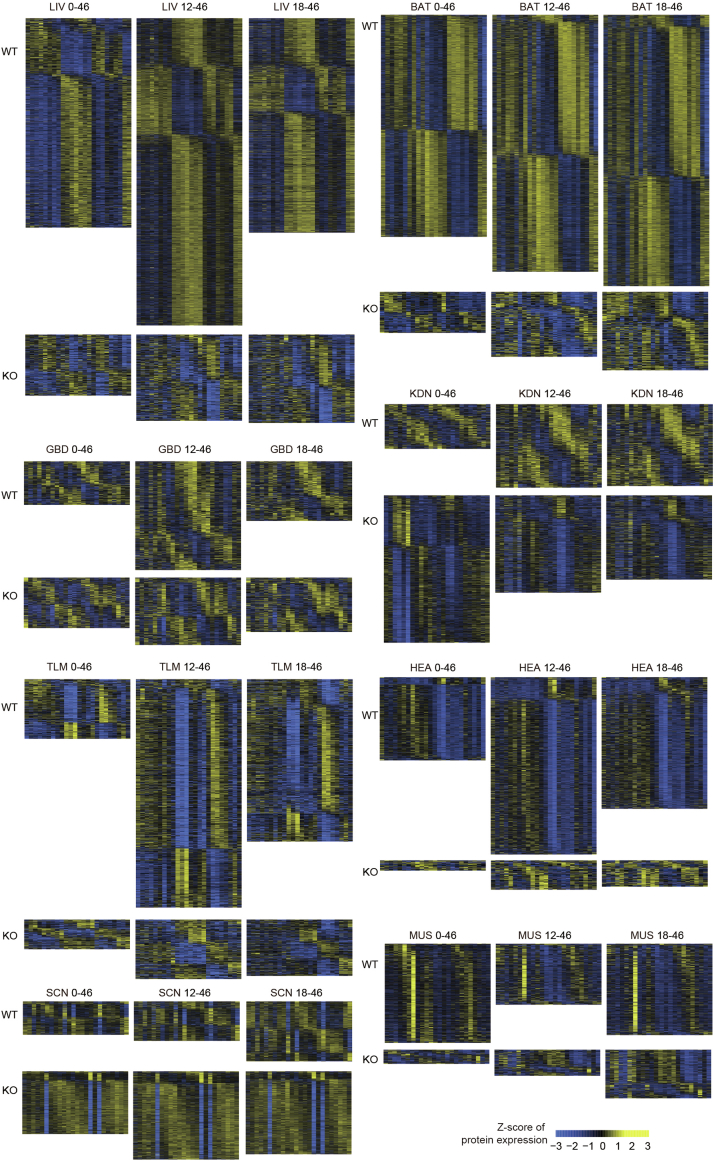
**Circadian proteome in different temporal intervals.** The heatmaps for the rhythmic proteins (meta2d_pvalue < 0.1 and rAMP > 0.1) during T0 to T46, from T12 to T46, and from T18 to T46 in WT and DKO mice. SCN indicates the SCN-containing region.

When studying the circadian transcriptome, it is common practice to exclude the first 18 h from the analysis (9, 46). However, it remains unclear whether this exclusion is relevant for protein levels, and little evidence has been provided regarding the impact of changes in the LD cycle on the rhythmic proteome under endogenous circadian control. To address this, we examined the circadian proteome in three different temporal intervals: from T0 to T46, from T12 to T46, and from T18 to T46 (supplemental Fig. S3 and supplemental Table S3).

In five out of eight tissues (LIV, HEA, KDN, TLM, and GBD), we observed that most protein cyclers were identified within the circadian proteome data collected between T12 and T46. In two tissues (BAT and SCN), most cyclers were observed in the circadian proteome from T18 to T46, while for the MUS, most cyclers were identified from T0 to T46. Consequently, we focused on the proteome data collected between T12 and T46 to investigate the rhythmic proteome under endogenous circadian control (supplemental Tables S1–S3).

**Figure S4:**
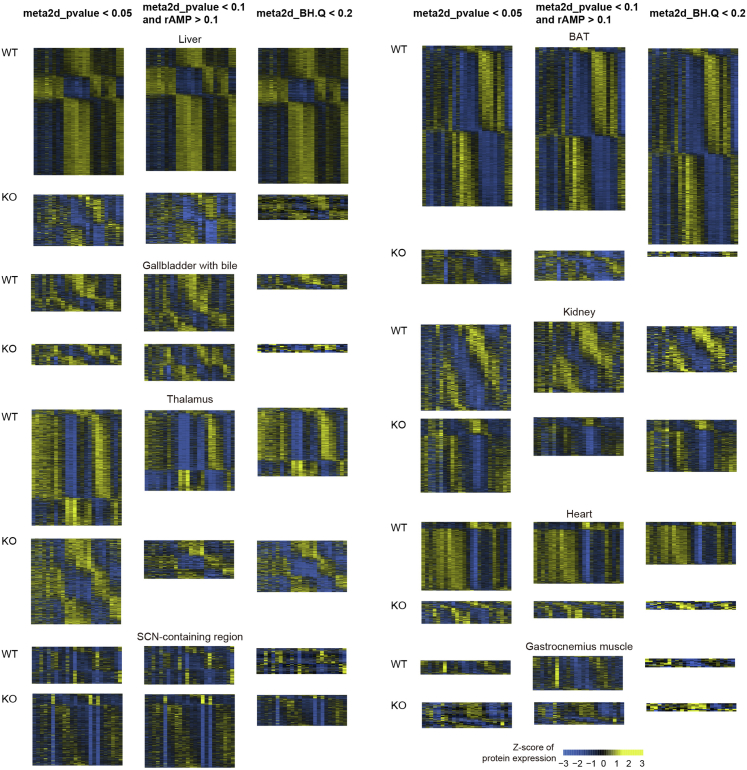
**Circadian proteome by three different thresholds.** The heatmaps for the rhythmic proteins by meta2d_pvalue < 0.1 and rAMP ≥ 0.1, meta2d_pvalue < 0.05, and meta2d_BH.Q < 0.2 from T12 to T46 in WT and DKO mice. SCN indicates the SCN-containing region.

Using MetaCycle (41), we identified 4749 rhythmic proteins in the WT mice. The fraction of cycling proteins ranged from 3.9% (SCN-containing region) to 30.2% (BAT) (Fig. 1*B* and supplemental Table S3). While in the DKO mice, only 1973, less than half the number of the WT, were identified (Fig. 1*B*, supplemental Fig. S4, and supplemental Table S3). These findings highlight the extensive control exerted by PER1 and PER2 on the circadian proteome, consistent with the observed arrhythmic behavior (4).

However, in SCN-containing region, we identified 325 rhythmic proteins in the WT mice, while 727 were identified in the DKO ones (Fig. 1*B*, supplemental Figs. S3 and S4, and supplemental Table S3). We found that proteins involved in mitochondrial metabolism and molecule transport, such as organophosphate ester transport, gained rhythmicity in the SCN of DKO mice (supplemental Table S4).

**Figure S5:**
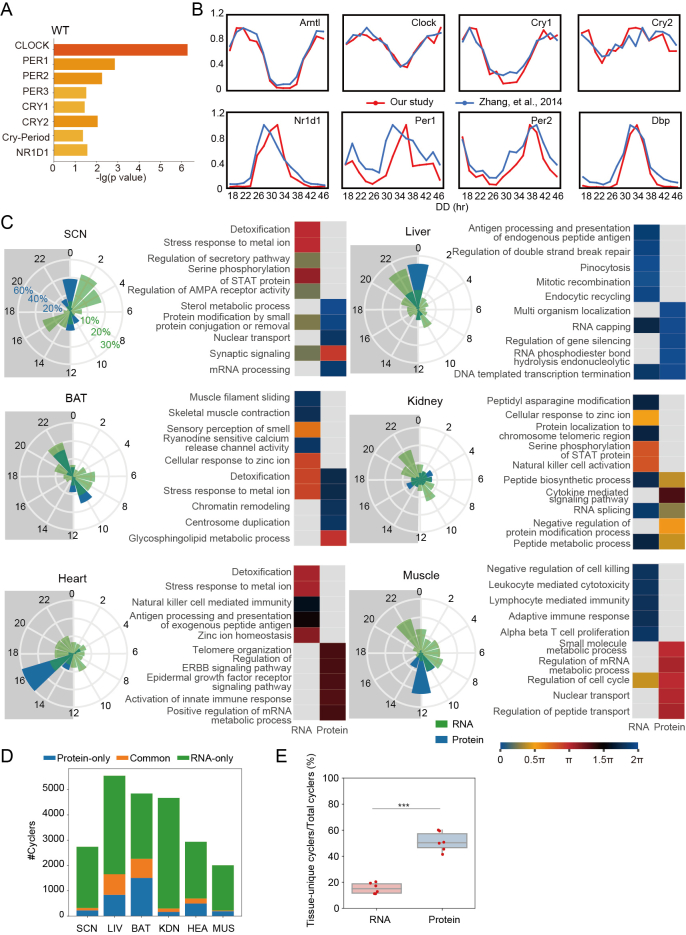
**Rhythmic expression of the common protein cyclers and comparison of the pathways enriched using PSEA between the two mice models, Related to Figures 2, 3.***A*, Core-clock proteins enriched using the upstream regulator analysis tool (in IPA) on the protein cyclers from the WT mice. None were enriched in the DKO mice. *B*, the time-series expression of core clock genes in liver samples of WT mice assayed by RNA-seq in our study and microarray in the previously published study. The gene expression levels were normalized by their ratio to the maximum value at all time points. *C*, the radial plots represent the distribution of the peak phases of the rhythmic mRNAs and proteins in seven tissues. The proportions of the cyclers for each circadian time are listed in the radar plot. The heatmaps compare the top five enriched pathways, returned by PSEA, between the two mice models in each tissue. *D*, the bar plot represents the protein-unique, mRNA-unique, and common cyclers based on the genes quantified both in the mRNA and the proteins dataset. *E*, pair-wise comparison of the proportions of the tissue-specific cyclers in seven tissues, based on the genes quantified both in the mRNA and the proteins dataset, performed using Student’s *t*-test. ∗∗∗, *p* value < 0.001.

In our study, we quantified nine out of the eleven known core clock proteins of the mouse TTFL (47), all of which were cycling in at least one tissue from the WT mice (Fig. 1*C*). By contrast, in the DKO mice, only BMAL1 and NR1D1 were observed to cycle (Fig. 1*C*), indicating that the circadian clock of the DKO mutants almost lost its rhythmicity. To further investigate the regulation of the rhythmic proteome, we employed the upstream regulator analysis tool from the IPA. This analysis revealed that in the WT mice, the rhythmic proteome was governed by seven core clock proteins, namely CLOCK, three PER proteins, two CRY proteins, and NR1D1 (supplemental Fig. S5*A* and supplemental Table S3). However, deficient TTFL regulation was observed in the DKO mice.

These findings suggest that (i) rhythmicity of the core clock proteins are almost abolished in the DKO mice; (ii) the TTFL controls a large portion of the circadian proteome; (iii) the circadian proteome exhibits tissue-specific patterns.

### Proteome Rhythm Reflects Circadian Anticipatory Phenomena

To compare the phase distribution between proteome rhythm and transcriptome rhythm across multiple tissues, we firstly examined the dynamic expression of core clock transcripts among the liver samples collected for proteomic assay and found that the phases of these core clock transcripts in our study were almost identical to those in the previously published circadian transcriptome dataset (GSE70384 in Gene Expression Omnibus) (9) (supplemental Fig. S5*B*). This similarity indicates that both datasets were conducted under similar feeding environment and experimental settings. The previously published dataset also collected tissue samples at 2-h interval, just like our study. Additionally, six of the twelve tissue types analyzed in the published dataset overlapped with our study, namely SCN-containing region, liver, BAT, gastrocnemius muscle, kidney, and heart. We thus chose to employ the previous dataset into our study.

We observed that only a few proteins and transcripts exhibited cycling patterns at both levels in WT mice (Fig. 2*A*). By comparing the phase differences of these common cyclers using CircaCompare (42), we found that nearly all of them peaked at similar circadian time without any statistically significant difference (supplemental Table S3). However, most protein cyclers did not exhibit cycling patterns at the cognate mRNA level. In the liver, for instance, 45% of the protein cyclers also cycled at the mRNA level (Fig. 2*A*), whereas in gastrocnemius muscle, only 10% of the protein cyclers exhibited oscillations at the RNA level (Fig. 2*A*). This observation not only highlights the tissue specificity of the circadian clock but also suggests that more than half, up to 90%, of the protein cyclers are independent of transcript rhythm. This can be attributed to various factors, such as translational and posttranslational regulation, protein turnover, as well as noise in the analysis.

We next proceeded to compare the phase distributions of all protein and RNA cyclers in the WT mice. We found that the two distributions were significantly different in all tissues (Fig. 2*B* and supplemental Fig. S5*C*). Interestingly, the peak phases of the cycling proteins in the WT mice primarily clustered within two narrow windows at the LD transition (Fig. 2*B*). In contrast, the cycling mRNAs exhibited a relatively uniform distribution, with the peak at dusk (the period around T12) diminished and the peak at dawn (the period around T0) shifting approximately 6 h earlier compared to the protein cyclers (Fig. 2*B*). This result indicates that in WT mice, the proteomic oscillations are more robust, compared to transcriptomic oscillations, in anticipation of the beginning of the activity and rest phases during the LD cycle.

In the SCN-containing region, LIV, BAT, and KDN, two phase peaks were observed with different phase shifts between mRNA and protein levels (Fig. 2*B*). In HEA, although the oscillations of mRNA cyclers peaked across circadian time, protein cyclers exhibited a prominent peak phase at inferred activity phase (Fig. 2*B*), which might be associated with increased energetic demand, as previously reported (48, 49). In MUS, mRNA cyclers peaked from the end of inferred activity phase throughout the inactivity phase, whereas protein cyclers exhibited intensive peaks during the period of transition from the rest to activity phases (Fig. 2*B*), indicating that the protein rhythm in the MUS peaks in the anticipation of active period.

Comparing the phase distribution of cycling proteins in the DKO mice to that of the WT mice, we observed a different pattern (Fig. 2*B*). In the DKO mice, the peak phases of different tissues exhibited a dispersed distribution, and only a single, broad peak was detected across all tissues between T16 and T24. Notably, the striking disappearance in the DKO mice of those protein cyclers that peaked at T0 and T12 in the WT mice suggests the PER1/2 may control the anticipation of the circadian rhythm.

### More Protein Cyclers Exhibit Striking Tissue Specificity

We next investigated the tissue specificity of cyclers in the WT mice. On average, 47.7% of the protein cyclers were tissue-specific—a significantly higher percentage than the mRNA cyclers had shown (19.9%) (Fig. 3*A* and supplemental Table S3). To ensure that this difference was not influenced by varying gene expression levels across the eight tissues, we also analyzed the tissue-specific cyclers based on the genes quantified at both the transcriptome and the proteome levels within each tissue. Even with this normalization, we still found a significantly higher proportion of tissue-specific protein cyclers than mRNA cyclers in each tissue (supplemental Fig. S5, *D* and *E*). This indicates that the higher degree of tissue-specific protein rhythm is unlikely to be solely attributed to the higher number of tissue-specific proteins (Fig. 1*B*). Furthermore, when comparing the number of cyclers common to multiple tissues, we observed that 1170 mRNA but only 13 protein cyclers were shared among four tissues (Fig. 3*B*), while the numbers decreased to 476 for mRNA and zero for protein cyclers when comparing more than four tissues (Fig. 3*B*). These observations taken together consolidate involvement of translational and posttranslational regulation in the tissue-specific rhythmicity.

**Figure S6:**
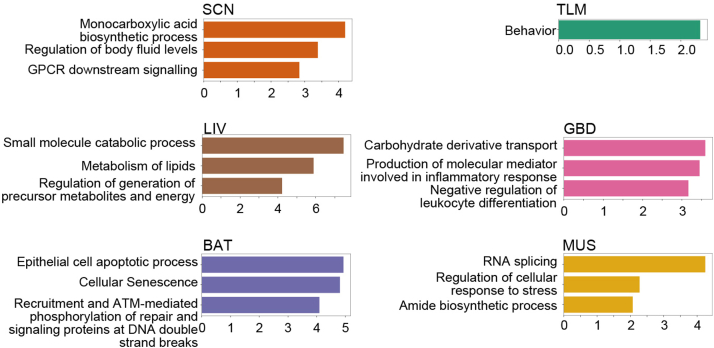
**Enriched pathways for cyclers with different rAMPs between the WT and the DKO mice, Related to Figure 3.** The bar plots represent the three most enriched pathways for high-rAMP and tissue-specific cyclers (in the WT mice). SCN indicates the SCN-containing region.

To eliminate the influence of intrinsic noise in protein expression and underline the biological significance of rhythmicity, we focused on cyclers with a high rAMP (rAMP >0.3) (50). By increasing the rAMP threshold, the proportions of tissue-specific cyclers in both the WT and the DKO mice were significantly increased (Fig. 3*C* and supplemental Table S3). Among the cyclers with high rAMP, 79.6% and 95.3% (tissue-specific cyclers over total cyclers of each tissue) were tissue-specific in the WT and the DKO mice, respectively (Fig. 3*C* and supplemental Table S3). Taking the liver as an example, in the WT group, a total of 167 high-rAMP cyclers were identified, out of which 120 were specific to the liver. In the case of the DKO group, 14 out of 17 high-rAMP cyclers were found to be specific to liver tissue (supplemental Table S3). In the WT mice, the tissue-specific cyclers with high rAMP were also involved in the main processes of the respective tissues, such as the behavior in TLM and metabolism in LIV (supplemental Fig. S6, *A* and *B* and supplemental Table S4).

**Figure S7:**
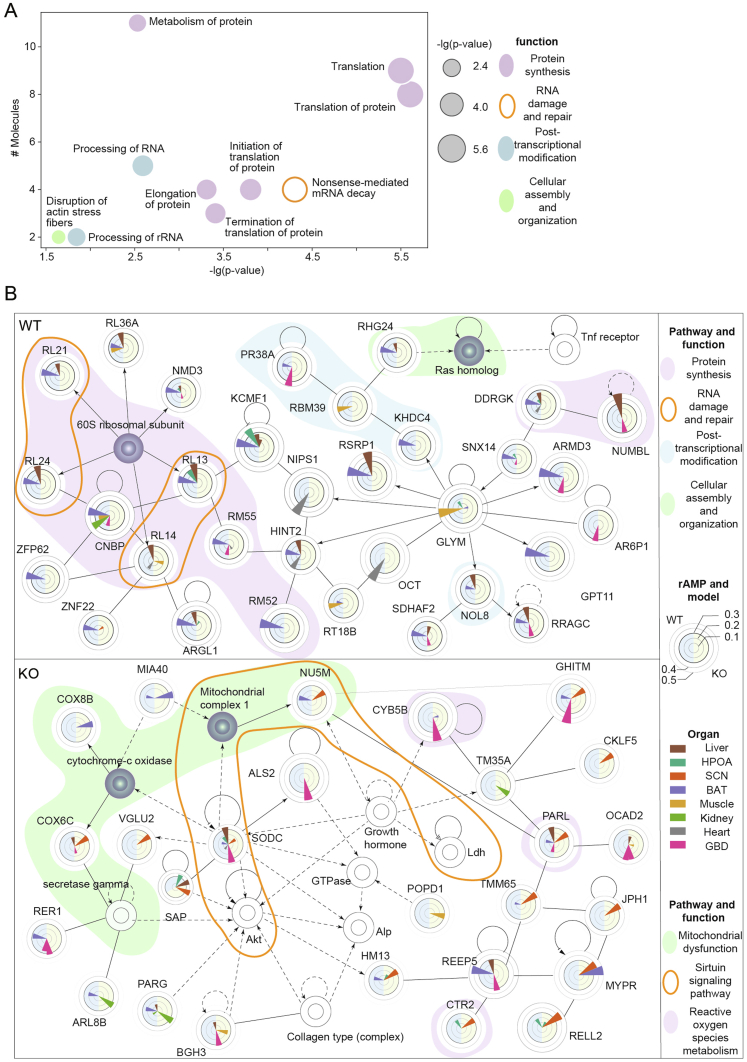
**Enriched network of the high rAMP cyclers, Related to**Figure 3**.***A*, enriched functions of the high rAMP cyclers from WT mice. *B*, networks were enriched for high rAMP cyclers using IPA (score > 0.5). The pathways and the functions of the cyclers in the network were annotated using IPA. SCN indicates the SCN-containing region.

By IPA, we identified several high-rAMP cyclers that specifically cycled in the WT mice and were involved in protein synthesis, RNA damage and repair, posttranscriptional modification, and cellular assembly and organization (supplemental Fig. S7, *A* and *B* and supplemental Table S4). Examples of proteins involved in these posttranscriptional processes included the 60S ribosomal protein subunits (RL13, RL14, RL21, RL24, and RL36A), the 39S ribosomal protein subunits of the mitochondria (RM52 and RM55), the pre-mRNA splicing factors associated proteins (PR38A and RBM39), and the mRNA splice site selection–associated protein (KHDC4) (supplemental Fig. S7*B*). On the other hand, high rAMP cyclers in the DKO mice were associated with mitochondrial dysfunction, sirtuin signaling pathways, and reactive oxygen species metabolic process (supplemental Fig. S7, *A* and *B* and supplemental Table S4). Thus, the different circadian behaviors of our two mice models may, at least partially, have been driven by these pathways.

### Protein Cyclers Exhibit Higher Degree of Tissue Specificity than Transcript Cyclers

To gain insights into the functions and phases of protein cyclers in the WT mice, we conducted PSEA to identify well-synchronized pathways enriched for cyclers with nonuniformly distributed phases and highly temporal cohesiveness at a specific circadian time (43) (supplemental Table S5). We compared pathways shared by both mRNA and protein cyclers in each tissue (supplemental Fig. S5*B* and supplemental Table S5) and found a six- to ten-hour phase shift in most of these pathways. In addition, we found that more than half of the top five well-synchronized pathways exhibited tissue specificity at both the mRNA (14/26) and protein (16/29) levels (Kuiper’s *p* value (vs. background) < 0.05, Kuiper’s q-value (vs. uniform) < 0.05, ranked by vector-average magnitude) (Fig. 3*D* and supplemental Table S5).

Furthermore, among the shared pathways among different tissues, we found 10 pathways enriched at almost inverted phases (more than 10-h phase shift) for cycling proteins, while only three were at inverted phases for cycling mRNAs (Fig. 3*E* and supplemental Table S5). This indicates that shared pathways for cycling proteins exhibit more diverse phases among different tissues than cycling mRNAs. Notably, these pathways with heterogeneous phases were primarily involved in posttranscriptional, translational, and post-translational processes, such as RNA metabolism, nuclear transport, and protein modification. These findings suggest that tissue-specific rhythmicity at the proteomic level may be attributed to these processes.

### Tissue-Specific Protein Cyclers in the WT Mice are Mainly PER-Dependent

**Fig. 4:**
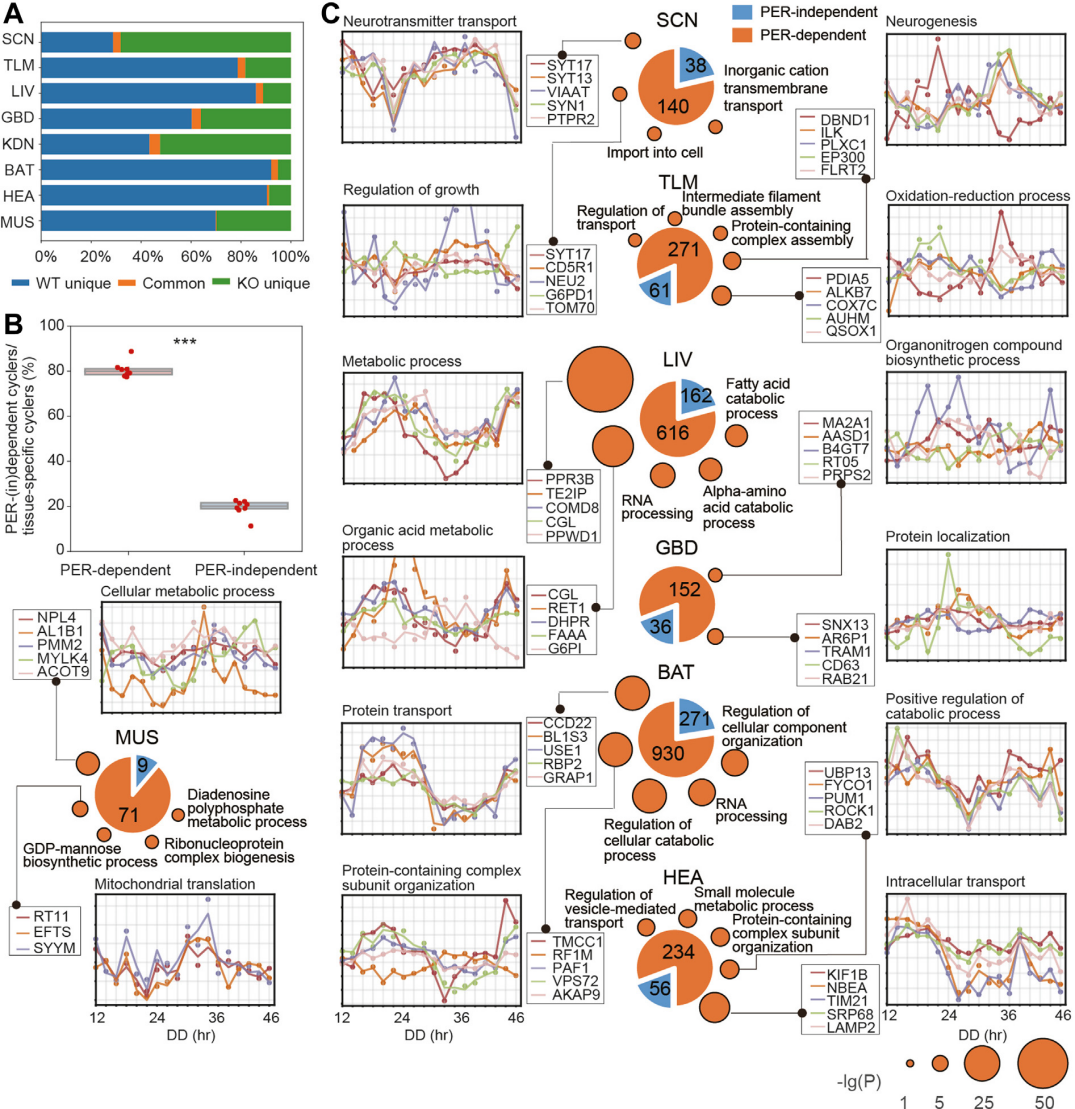
**PER-dependent and tissue-specific cyclers and their involved pathways in the WT mice.***A*, proportions of the WT-unique, the DKO-unique, and the shared cyclers over the total cyclers in each tissue. *B*, the PER-dependent and PER-independent cycler proportions of the tissue-specific cyclers. *p* value was computed using a paired Student’s *t* test. ∗∗∗, *p* value <0.001. *C*, tissue-specific and PER-dependent cyclers with their functions. The pie plots represent the numbers of the tissue-specific, PER-dependent, or PER-independent cyclers from each tissue. The circles around the pie plots represent the enriched pathways for the tissue-specific and PER-dependent cyclers (analysis performed using Cytoscape). Redundant pathways were removed by redundancy cutoff <0.1 and manual check. The area of the circles represents -lg(*p* value). The time-series curves represent the rhythmic expression of the top five cyclers with minimal *p* values (computed with MetaCycle) in the top two pathways. SCN indicates the SCN-containing region. DKO, double knockout; SCN, suprachiasmatic nuclei.

**Figure S8:**
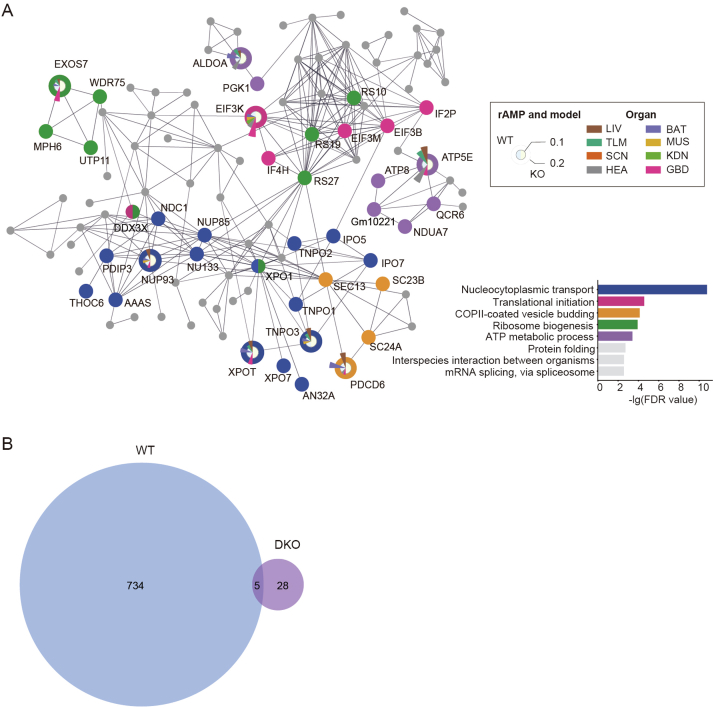
**The comparison and correlation of protein cyclers between WT and DKO mice.***A*, interactions between the protein cyclers rhythmically expressed in more than two tissues from the WT mice but none from the DKO mice, Related to Figures 3 and 4. The network was generated using String and Cytoscape. The Gene Ontology process enrichment analysis was performed using Cytoscape. Redundant pathways were removed by redundancy cutoff < 0.1 and manual check. The top five pathways were selected by P value, and their corresponding cyclers were accordingly colored. The cyclers shared by at least four tissues in the WT mice are represented by a radar chart; the diameter of each sector represents the rAMP of the cyclers from the corresponding tissues. *B*, overlap of temporally correlated protein cyclers between the WT and DKO mice, Related to Figure 7. SCN indicates the SCN-containing region.

Next, we investigated whether the tissue-specific protein cyclers are still controlled by PERs. Over 90% of the cyclers found in the WT mice were PER-dependent (Fig. 4*A* and supplemental Table S3). Among the PER-dependent cyclers, a small group of 256 cyclers was shared by at least three tissue types (supplemental Fig. S8*A*, supplemental Tables S3, and S4), while 2516 cyclers were tissue-specific (supplemental Tables S3 and S4). In other words, the PER-dependent tissue-specific cyclers far exceeded the number of PER-independent ones (Fig. 4, *B* and *C*).

The shared cyclers that were PER-dependent and present in multiple tissues primarily involved in essential cellular functions, such as translation, energy metabolism, and cytoskeleton organization (supplemental Fig. S8*A*, supplemental Tables S3, and S4). On the other hand, the PER-dependent tissue-specific cyclers were involved in specific processes such as neurotransmitter transport in SCN-containing region, neurogenesis in thalamus, and metabolic processes in liver, BAT, and gastrocnemius muscle (Fig. 4*C* and supplemental Table S4). These tissue-specific cyclers were associated with the main functions of their respective tissues, indicating their importance in tissue-specific processes (Fig. 4*C* and supplemental Table S4).

Interestingly, most cyclers belonging to the same pathway were found to oscillate with a similar phase, indicating synchronization among tissue-specific cyclers (Fig. 4*C*). Here are a few relevant examples. The first is SYT17 and SYT13, two subunits of synaptotagmin, a Ca^2+^ sensor that triggers neuronal exocytosis (51). Both were found to be SCN-specific, PER-dependent, and peaking at around T12 (Fig. 4*C*). Another example is vasopressin-neurophysin 2-copeptin (NEU2), which is mainly synthesized in TLM magnocellular nuclei. It is cleaved to vasopressin before being secreted into the circulatory system. Vasopressin is involved in osmoregulatory and central nervous effects (52). Our data showed NEU2 specifically cycled in SCN-containing region with high rAMP peaking at around T11 (Fig. 4*C* and supplemental Fig. S6*B*) and may regulate the circadian rhythms of the fluid balance and the vascular tone systemically. Multiple protein cyclers involved in the metabolism of glycogen and amino acid, including dihydropteridine reductase, fumarylacetoacetase, and protein phosphatase 1 regulatory subunit 3B (PPR3B), were liver-specific and PER-dependent, peaking at around T20 (Fig. 4*C*). Finally, two proteins involved in mitochondrial translation (53), namely 28S ribosomal protein S11 (RT11) and elongation factor Ts, were both specifically cycling in MUS of the WT mice and peaked at around T9 (Fig. 4*C*). Taken together, our data suggest that most of the tissue-specific cyclers involved in well-synchronized pathways are mainly controlled by PER1 and PER2.

### Diminished Oscillations in Nucleotide Excision Repair in DKO Mice

**Fig. 5:**
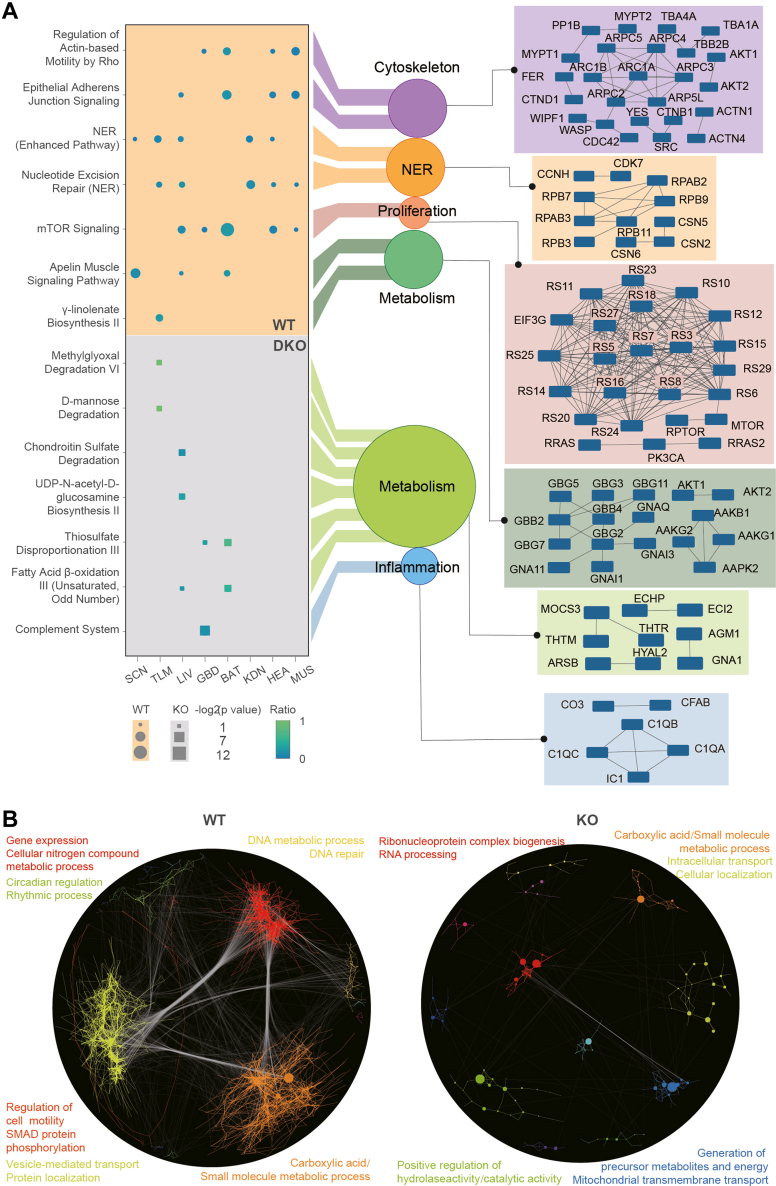
**Enriched pathways based on cycling proteins from the WT and DKO mice.***A*, mouse model–specific pathways across eight tissues and networks of the cyclers from each biological function. The *circle* and *square* represent the pathways enriched in the WT and the DKO mice, respectively. The size represents the statistical significance within the enriched pathways computed by Ingenuity pathway analysis. *B*, networks by Rcy3 and enriched pathways for each cluster of robust protein cyclers (*p* value <0.01 computed with meta2d, JTK, and ARS). The top two pathways from each cluster (with the minimal *p* values) are highlighted in the same color as its corresponding network cluster. SCN indicates the SCN-containing region. DKO, double knockout; SCN, suprachiasmatic nuclei.

To further investigate the PER-dependent pathways, we performed a pathway enrichment analysis of all the cyclers in the WT and DKO mice using IPA (supplemental Table S4). We found that the pathways associated with nucleotide excision repair (NER), proliferation, cytoskeleton organization, and metabolism were specifically enriched in the WT mice (Fig. 5*A*). To identify the most robust cyclers, we applied a strict cutoff (*p* value <0.01 by meta2d, Jonckheere-Terpstra-Kendall (JTK) and ARSER (ARS)). Even with this stringent filter, the WT mice still had significantly more cyclers than the DKO ones (1004 and 353, respectively) (Fig. 5*B* and supplemental Table S6). Using this set of robust cyclers, we observed that circadian regulation and rhythmic processes, as well as DNA repair, were only enriched in the WT mice (Fig. 5*B* and supplemental Table S6). It supports the finding that NER loses circadian rhythm in the DKO mice (54, 55).

**Fig. 6:**
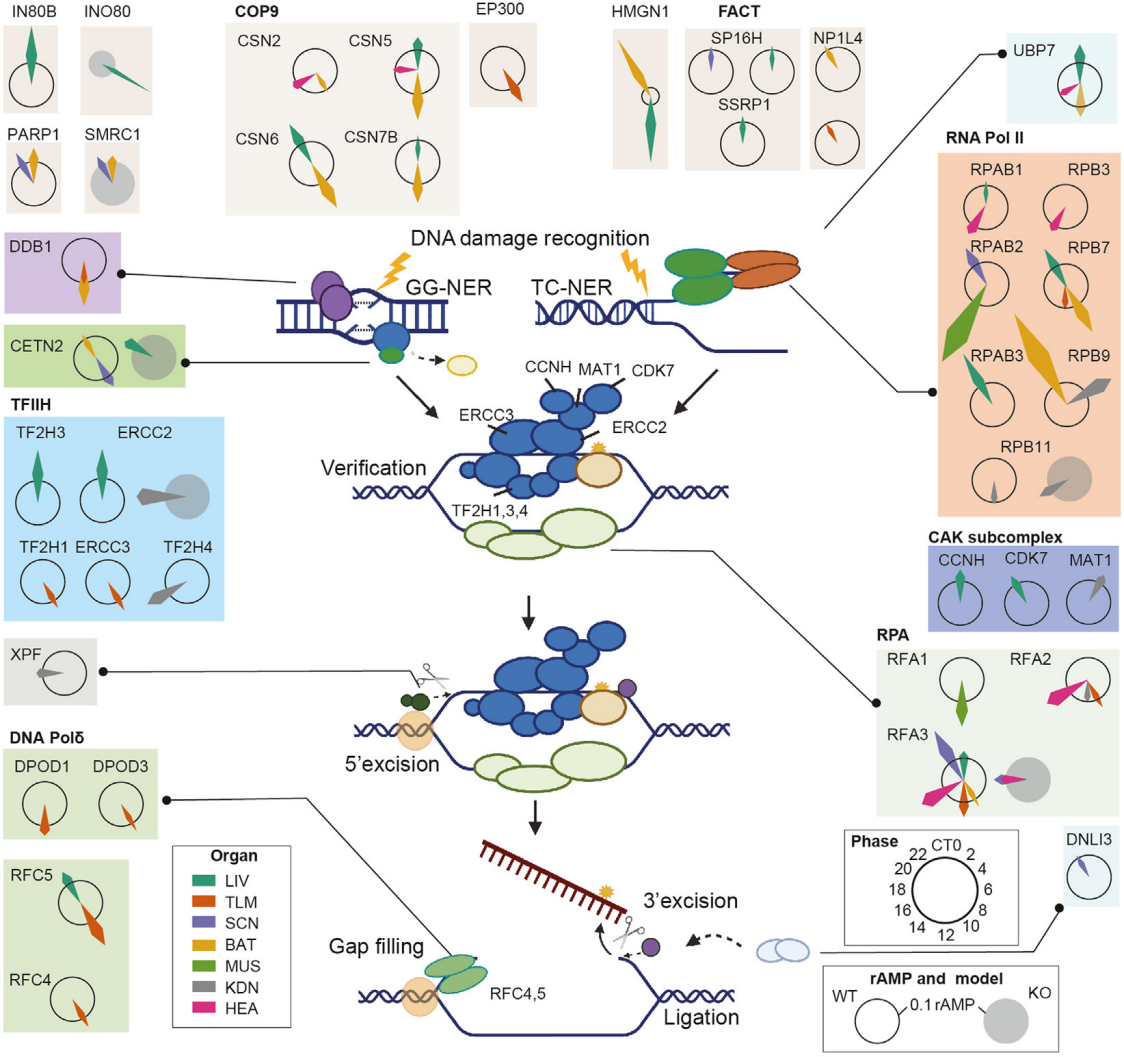
**The protein cyclers involved in the nucleotide excision repair pathway.** The key steps and proteins involved in the NER pathway are visualized. The protein cyclers involved in the NER pathway are further elaborated using radial plots. In these plots, the *hollow* circles and *solid gray circles* in radial plots represent cyclers in WT and DKO mice, respectively. The radial plots display the rAMP and the phase of the cyclers in the NER process, across eight tissues. The angles of the arrows indicate phases of protein cyclers. The colors of the arrows represent corresponding tissue types. SCN indicates the SCN-containing region. DKO, double knockout; GG-NER, global genome NER; TC-NER, transcription-coupled NER; NER, nucleotide excision repair; rAMP, relative amplitude value; SCN, suprachiasmatic nuclei.

Among the PER-dependent pathways enriched in the WT mice, NER was enriched in most of the brain and the peripheral tissues (Fig. 5*A*). NER is the main mammalian pathway for removing large DNA lesions (56) (Fig. 6). We found that 38 out of 45 cyclers involved in various steps of the NER pathway were specifically rhythmic in the WT mice (Fig. 6). Only RPB11 (in the kidney) and RFA3 (in the heart and SCN-containing region) were found to be cycling in both the WT and DKO mice (Fig. 6). Interestingly, cyclers belonging to the same complex, such as TFIIH, the CAK subcomplex, COP9, and others involved in similar functions, all peaked at a similar phase, whereas cyclers from different tissues showed a high degree of variation (Fig. 6). For example, the four subunits of COP9 in BAT, which regulate the initiation of global genome NER through its deneddylation activity (56), peaked between T10 and T12, while those in liver peaked between T22 and T0 (Fig. 6). The subunits of the TFIIH complex peaked at T0 in liver and at T10 in TLM (Fig. 6). The subunits of DNA Polδ and the replication factors C, which are involved in the final DNA gap-filling synthesis and ligation, all peaked between T10 and T12 in the TLM (Fig. 6). These findings suggest tissue-specific regulation and coordinated rhythmicity within the NER pathway.

### Intertissue Correlations of Circadian Proteome Revealed Severe Temporal Dissonance in the DKO Mice

**Fig. 7:**
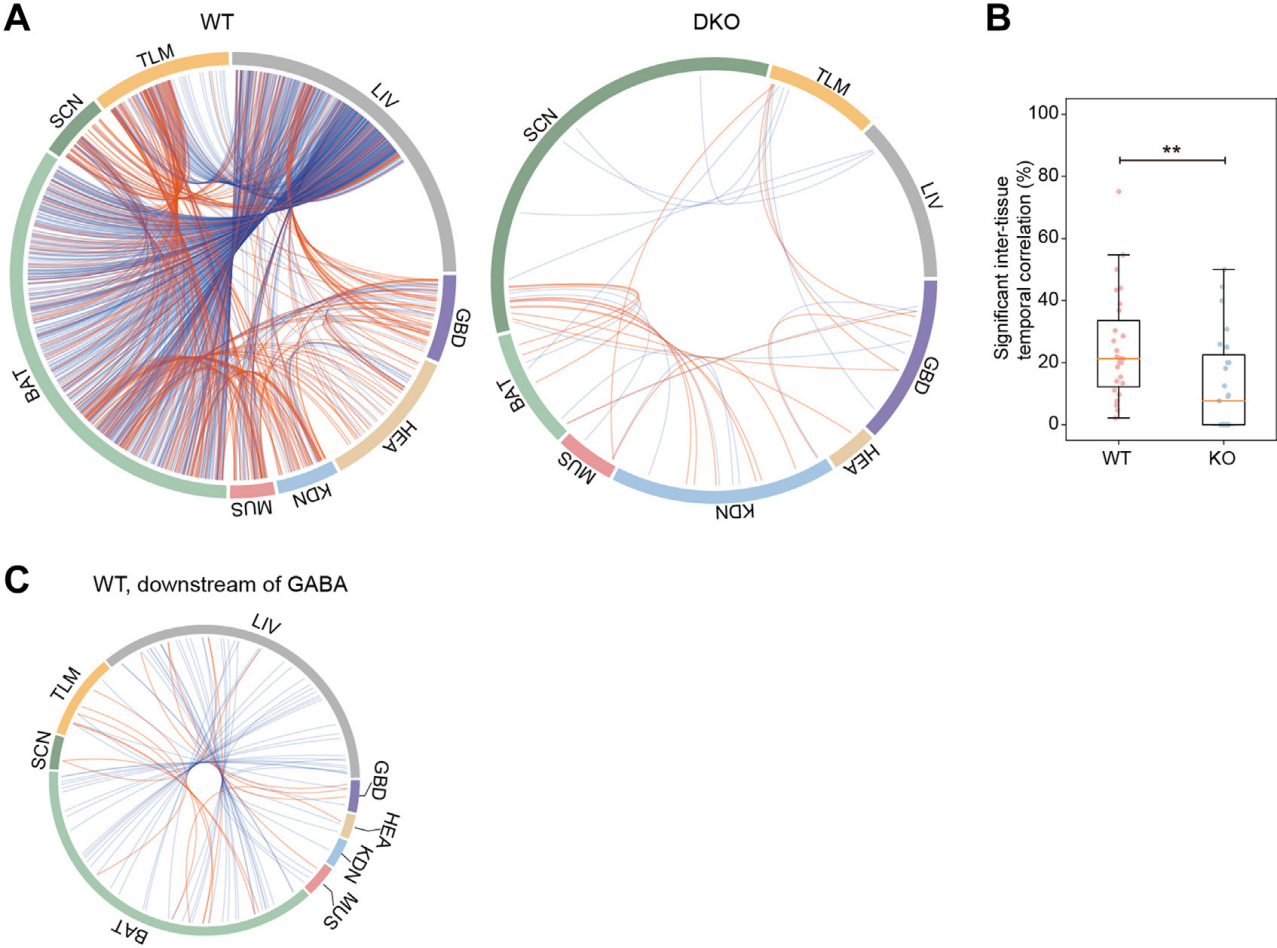
**Intertissue correlations of circadian proteome.***A*, intertissue temporal correlation of protein cyclers in the WT and DKO mice. The length of the colored arc in the outer ring represents the number of protein cyclers in each tissue. Each ribbon represents a significant intertissue correlation of the protein cycler (positive, *red*; negative, *blue*). *B*, the ratios of significant intertissue temporal correlation in each tissue between the WT and DKO mice. *p* value was computed using a paired Student’s *t* test. ∗∗, *p* value <0.01. *C*, intertissue temporal correlations of GABA-regulated downstream protein cyclers in the WT mice. The length of the colored arc in the outer ring represents the number of temporally correlated protein cyclers (Spearman’s rank correlation, *p* value <0.05) in each tissue. Each ribbon represents a significant intertissue correlation of the GABA-regulated downstream protein cycler (positive, *red*; negative, *blue*). SCN indicates the SCN-containing region. DKO, double knockout; SCN, suprachiasmatic nuclei.

Considering the pronounced tissue-specificity observed in the circadian proteome, we next asked how different organs and tissues synchronized their rhythmicity. To investigate this, we performed Spearman’s rank correlation analysis using our temporal proteome data to identify significantly correlated cyclers between each pair of tissues (Fig. 7). With a *p* value threshold of 0.05, we identified 739 and 33 cyclers with significant temporal correlation in the WT and DKO mice, respectively, with only five of them were shared by both mouse models (Fig. 7*A*, supplemental Fig. S8*B*, and supplemental Table S7). In addition, significantly higher ratios of cyclers with temporal correlation in each tissue of the WT mice (24.7%) were observed compared with that of the DKO mice (12.5%) (Fig. 7*B*). These indicate a severe intertissue temporal dissonance of circadian proteome in the absence of *Per1* and *Per2*. Then, we focused on the cyclers with positive temporal correlation and found that in the WT mice, these cyclers were involved in protein ubiquitination, autophagy, ferroptosis, nucleus-cytoplasmic transport, and circadian rhythm (supplemental Table S7).

To investigate potential synchronizers for these cyclers with temporal correlations in the WT mice, we performed upstream regulator analysis by IPA and found that γ-aminobutyric acid was the most significantly enriched upstream regulator (supplemental Table S7), which was annotated to regulate 50 temporally correlated cyclers (Fig. 7*C*). γ-Aminobutyric acid has been previously reported to be involved in the synchronization of circadian clock neurons (57) and coupling of regional pacemakers within SCN (58). In our data, it might play a role in intertissue correlations of circadian proteome, which needs further validation.

## Discussion

This study systematically investigated circadian rhythm of multiple organs and tissues on the proteome level. To enhance accessibility to this valuable resource, we have developed a web application with an interactive interface (https://prot-rhythm.prottalks.com/), which provides convenient access to all the data sets and statistical analyses. The major findings are (1) reduced protein cyclers in most peripheral tissues of DKO mice; (2) protein rhythm recaptures physiological functions of the respective organs and tissues, highlighting additional biological functions that are not seen in the transcriptome; (3) protein rhythm exhibits high degree of tissue specificity which is less obvious on the mRNA level; (4) PER1/2 control the circadian rhythm of a number of key proteins and pathways including NER; (5) multitissue circadian proteome in DKO mice exhibits severe temporal dissonance in intertissue correlations.

Compared with previous proteomic investigation of circadian rhythm which are exclusively limited to mouse liver tissue or *ex vivo* cell cultures (6, 7, 18, 19, 59), our study analyzed eight tissues in both the WT and *Per1*/*Per2* DKO mice. Despite the crucial roles of PER1/2 in circadian rhythm, the molecular consequence of *Per1/Per2* DKO has not been reported on proteome level. While the published proteomic studies analyzed up to about 6000 proteins (7, 60), our study covers over 11,000 proteins, allowing identification of proteins participating in circadian rhythm which were previously excluded from the analysis.

It has been reported that arrhythmic feeding is one of the consequences of the knockout of core clock genes, including *Per1/2* (61), *Bmal1*, and *Cry1/2* (62). In addition, the knockout of core clock genes results in the loss of rhythm in other behaviors, such as sleep and exercise. In this study, we aim to cover the coupling influences by knockout of Per1/2.

Here, we uncovered 4749 and 1973 protein cyclers from the WT and the DKO mice, respectively. In most peripheral tissues of the DKO mice, we found that the number of rhythmic proteins remarkably decreased when compared to the WT mice. However, in the SCN of the DKO mice, we observed an increase in the number of rhythmic proteins compared to the WT mice, which poses an intriguing finding. Recognizing that the SCN tissues inherently synchronize with the LD cycle, we have considered the plausible influence of the TTFL in suppressing the emergence of LD-driven rhythmic proteins. Our hypothesis stems from the notion that the disruption of TTFL could contribute to the observed rise in the count of diurnal proteins in the SCN. In this context, we speculate that if sustained darkness might result in the attenuation or disappearance of these rhythms in the SCN of DKO mice. However, it is important to note that we currently lack concrete evidence to support this proposition in the present phase of our research. Further investigation and experimental verification are required.

We also observed highly anticipatory bouts of circadian proteome in the WT mice, while the findings in circadian transcriptome were not as significant as the ones at protein level. In addition, the knockout of *Per1* and *Per2* abolished the anticipation of circadian proteome. Similarly, food anticipatory activity has been reported to be lost in the *Per2* mutant mice (63). The resulting well-synchronized biological processes executed by highly anticipatory protein cyclers, including the synaptic signaling in SCN-containing region and thalamus, detoxification in TLM, and BAT, likely facilitate the adaptation of mice to the daily environmental cycles (supplemental Table S5). In addition, the circadian proteome of the WT mice exhibited a higher tissue specificity than circadian transcriptome. This high tissue specificity maintained in the high-rAMP cyclers after excluding the noise of low-rAMP cyclers. The tissue-specific cyclers, in particular, were dependent on PER1 and PER2. These PER-dependent and tissue-specific protein cyclers are involved in the main physiological functions of each organ and tissue. Meanwhile, pathways such as NER and proliferation seemed to have weakened their circadian rhythm in the DKO mice. The intertissue temporal correlations of circadian proteome were extensively reorganized and severely dissonant in the DKO mice.

### Insights into Chronochemotherapy

NER has been reported as the only mechanism of DNA repair tightly controlled by the circadian rhythm (64). In the absence of NER, damage-induced mutations and chromosomal aberrations increase the risk of developing cancer, and damage-induced transcription arrests affect the cellular homeostasis and promote aging (56). Here we found that the oscillation of NER is controlled by PER1/2 (Figs. 5 and 6). Consistently, *Per2* mutant mice have been linked to a higher frequency of gamma radiation–induced tumorigenesis, which is mediated by the DNA damage pathways (65). We found that most cyclers from the NER pathway were enriched in TLM and liver. Rhythmic NER has also been reported in the mouse brain exposed to ultraviolet radiation and in cisplatin-exposed mouse liver (54, 64). In the brain, oxidative phosphorylation, the main energy source of neuronal activity, produces excess reactive oxygen species leading to DNA damage. NER’s activation is critical at peaks of neuronal activity, which is known to cycle along the circadian day. Moreover, the two cycling subunits of DNA Polδ in SCN are, in fact, the main NER polymerases for nonreplicating cells, including the nonreplicating neuronal cells of adults (56).

In summary, we found that the key proteins of each step of the NER pathway exhibit a circadian rhythm in multiple brain and peripheral tissues and mainly peaked at the light transition phase, which might help guide cisplatin chronochemotherapy.

### Limitations of this Study and Outlook

SCN is a small, well-defined bilateral structure. The SCN-containing region was obtained by experienced operators. The purity of SCN has been confirmed by immunofluorescence and RT-PCR analysis of the strong expression of synaptotagmin10 (Syt10) and time-series pattern of transcripts by core clock genes. Nevertheless, we could not exclude the impact of contaminants from tissues surrounding the SCN.

We have collected samples from 0 to 46 h. Through a segment-by-segment moving analysis of the 0 to 46 h data, we found that the data between 12 to 46 h was relatively stable across tissues. Therefore, we only have proteomic data spanning 34 h in the downstream analysis. What we learned is that future proteomic studies of its kind should start from 18 h and end after 64 h.

Per1/2 DKO results in arrhythmic behaviors, such as feeding, exercise, and sleep; therefore it is difficult to distinguish the arrhythmic proteins driven by these behaviors. Thus, this study analyzed the compound influences on circadian proteome by knockout of Per1/2.

Another limitation of our study is that while the depth of proteomics analysis is higher than those in the literature (7, 18, 19, 60), but still some known core clock proteins are not identified in every sample. Future proteomic studies of higher depth are required. The proteome depth is lower than that of transcriptome studies (9). The transcriptome studies provide insights at the mRNA level, whereas our proteomics studies concerns proteins which are the major building blocks and driver of biochemical reactions.

We provided a rich resource of the circadian proteome atlas in both WT and DKO mice with MS-based proteomics technologies. However, validation by other technologies, such as Western blot, and by independent biological replicates is needed in the future.

In conclusion, our circadian proteome atlas revealed that protein oscillations regulated by posttranscriptional processes exhibited anticipatory and tissue-specific. These tissue-specific cyclers are mostly PER-dependent and directly involved in the main biological functions of the tissues they are originated from. The intertissue correlations of circadian proteome further revealed severe temporal dissonance in the DKO mice. We also found that the NER pathway was particularly enriched in cyclers of the WT mice but not the DKO. Our systemic analysis of the rhythmic proteins thus also provides valuable new insights into chronochemotherapy.

## Data Availability

All data are available in the manuscript or the supplementary material. The proteomics data are deposited in the iProX (IPX0003474000). Link: https://www.iprox.cn/page/SSV024.html;url=1694850915429X3je.

## Supplemental data

This article contains supplemental data.

## Conflict of interest

T. G. and Y. Z. are shareholders of Westlake Omics Inc; Y. L. W. G. and Q. Z. are employees of Westlake Omics Inc. All other authors declare that they have no conflicts of interest with the contents of this article.
